# The FGFR inhibitor pemigatinib overcomes cancer drug resistance to KRAS G12C inhibitors in mesenchymal lung cancer

**DOI:** 10.1371/journal.pone.0327588

**Published:** 2025-08-11

**Authors:** Angela Abdollahi, Margaret Favata, Michael Weber, Valerie Roman, Rodrigo Hess, Kayla Hammond, Matthew R. Farren, Mike Schaffer, Aidan Gilmartin, Hui Wang, Jonathan Rios-Doria, Alejandro Amador-Arjona

**Affiliations:** Incyte Research Institute, Wilmington, Delaware, United States of America; QUT Health: Queensland University of Technology Faculty of Health, AUSTRALIA

## Abstract

KRAS mutations are high prevalence oncogenic drivers for multiple cancers. With the advent of new classes of KRAS inhibitors that are showing meaningful clinical activity, research is now turning to questions of optimal combinations of therapies for specific indications, as many patients with KRAS G12C mutations do not respond and/or develop resistance to single-agent treatment. Here, we investigate combination therapies that may overcome resistance to KRAS G12C inhibitors. We found that pemigatinib, a potent and selective FGFR1–3 inhibitor, had a significantly high Bliss synergy score in combination with KRAS G12C inhibitors, and FGFR1 activity was shown to decrease KRAS G12C-dependency conferring inherent resistance in mesenchymal-like cell lines. Knockdown experiments verified the importance of FGFR1, but not FGFR2-4, for the synergistic effect with KRAS G12C inhibitors. Additionally, human lung cancer xenograft and patient-derived xenograft models with a mesenchymal phenotype and high FGFR1 expression were sensitive to the combination of G12C inhibitors and pemigatinib. In short, we demonstrate that pemigatinib and KRAS G12C inhibitors are promising agents for combination therapy in non-small cell lung cancer with a mesenchymal-like phenotype harboring high FGFR1 expression and KRAS G12C mutations to broaden patient response.

## Introduction

KRAS is the most frequently mutated oncogene in human cancer, accounting for roughly 30% of non-small cell lung cancer (NSCLC) cases [1]. KRAS is a guanosine triphosphatase (GTPase) that functions in the transduction of signals downstream from activated receptors at the cell membrane to promote migration, survival, and differentiation of the cell. When functioning normally, KRAS cyclically switches from the guanosine diphosphate (GDP)-bound inactive state to the GTP-bound active state, which activates downstream signaling [2]. Glycine to cysteine substitution of KRAS at amino acid 12 (G12C) is a gain-of-function mutation whereby the protein has reduced GTPase activity, resulting in constitutive activation of downstream signaling pathways and leading to tumorigenesis [2]. KRAS G12C occurs in approximately 12% and 3% of NSCLC and colorectal cancers (CRC), respectively, and less frequently in other solid tumors [3].

After decades of considering KRAS to be undruggable [4], the discovery and elucidation of binding sites enabling the KRAS G12C class of inhibitors represents an important break-through in the field of oncology. Clinical trials of the KRAS G12C inhibitor AMG 510 (sotorasib, Amgen) have shown promising results, with some patients experiencing significant tumor shrinkage or stabilization [5,6]. In May 2021, the US Food and Drug Administration (FDA) granted accelerated approval to AMG 510 for the treatment of metastatic NSCLC with the KRAS G12C mutation [7]. Several companies have since developed their own KRAS G12C inhibitors, including adagrasib (MRTX849, Mirati Therapeutics) [8,9], GDC-6036 (Roche) [10], and JDQ443 (Novartis) [11]. Despite impressive evidence of disease control in KRAS G12C-mutant NSCLC indicating clear target engagement, roughly one third to one half of patients did not experience objective response, suggesting a substantial population with incomplete KRAS G12C-dependency; moreover, many patients with KRAS G12C–mutant lung cancer develop resistance to single-agent KRAS G12C inhibitor treatment [12,13]. Studies in both preclinical models and post-treatment clinical samples suggest the reactivation of mitogen-activated protein kinase (MAPK) signaling and/or mutant KRAS-independent activation of the phosphatidylinositol 3-kinase (PI3K) pathway as common mechanisms resulting in KRAS G12C inhibitor resistance [14,15].

Adaptive feedback through multiple receptor tyrosine kinases (RTKs) drives resistance to KRAS G12C inhibition through compensatory activation of wild-type RAS genes, which bypasses KRAS-mediated signaling and cannot be inhibited by G12C-specific inhibitors [16]. Growing evidence has indicated that fibroblast growth factor (FGF)/receptor (FGFR) signaling plays a key role in resistance to multiple therapies [17]. The FGF family is part of the RTK family, comprising of 18 ligands, which exert their functions through four highly conserved tyrosine kinase receptors (FGFR1, FGFR2, FGFR3, and FGFR4) [17]. When FGF binds to its receptor, it activates the adaptor protein FGFR substrate 2 (FRS2), which then acts as a docking site for growth factor receptor bound protein 2 (GRB2). GRB2 is then able to trigger the RAS/RAF/mitogen-activated protein kinase–ERK kinase (MEK)/extracellular signal-regulated kinase (ERK) pathway, and to a lesser extent, the PI3K/protein kinase B (AKT) pathway. Several mechanisms of gene alterations are known to promote oncogenic FGFR signaling, including activating gene alterations through mutations, fusions and rearrangements, and gene amplifications. In addition, FGFR signaling can be elevated through overexpression of ligands and receptors through changes in the tumor or tumor microenvironment that promote epithelial-mesenchymal transition (EMT). FGFR1 expression has been demonstrated to be elevated in cancer cells that have undergone EMT and exhibit a mesenchymal phenotype [18]. EMT is a reversible mechanism of cellular re-programming, driven by both intrinsic and local extrinsic factors that alters the cancer epithelial cell traits, impacting gene expression, morphology, metabolism, proliferation, and migration. EMT promotes the progression of cancer by triggering loss of cell-cell adhesion, leading to a shift in cytoskeletal dynamics; the relationship between EMT and therapy resistance is well documented [19]. Hallmarks of EMT include the loss of E-cadherin expression and concomitant increase of mesenchymal markers, such as vimentin and N-cadherin [18].

Here, we report results from studies examining the combination of KRAS G12C inhibitors and pemigatinib. The activity of single-agent versus combination treatments was measured in cell viability assays, and screen hits were validated by in vitro mechanism of action studies as well as in vivo xenograft and patient-derived xenograft (PDX) models. We present the screening and functional studies that identified FGFR1 activation as a feedback mechanism following KRAS G12C inhibition in lung cancer cells with a mesenchymal phenotype. In addition, we demonstrate high levels of synergy between covalent KRAS G12C inhibitors and pemigatinib, a potent and selective FGFR1–3 inhibitor.

## Materials and methods

### Cell culture

KRAS G12C cell lines were cultured for analysis in the combination screen. All cell lines were obtained from American Type Culture Collection (ATCC; Manassas, VA), except LU99 cells obtained from the Japanese Collection of Research Bioresources Cell Bank (FUJIFILM Wako Chemicals U.S.A. Corporation, Richmond, VA) and HCC44 cells obtained from the German Collection of Microorganisms and Cell Cultures (Braunschweig, Germany). All cell lines were authenticated by short tandem repeat profiling. The following lung cancer cell lines were cultured in RPMI-1640 supplemented with 10% heat inactivated fetal bovine serum (FBS): NCIH1792, NCIH2122, NCIH358, NCIH2030, SW1573, HCC44, LU99. Calu-1 (lung cancer) cells were cultured in McCoy’s 5A Modified Medium and SW837 (colorectal cancer) cells were cultured in Dulbecco’s Modified Eagle Medium (DMEM). All cell lines were maintained in an incubator set to 37°C with 5% CO_2_, and all were negative for mycoplasma.

### Inhibitors

Pemigatinib was provided by Incyte Corporation (Wilmington, DE). RLY-4008 was purchased from MedChemExpress (#HY-147250). Drugs for in vitro validation were dissolved in dimethyl sulfoxide (DMSO) to yield a 5-mM stock solution and stored at −80°C. Inhibitory concentration assays and purity analyses were performed on all inhibitors before the screening assay.

### Drug combination screening

Screening was performed in 384-well, white-walled, clear-bottomed plates using the respective cell lines plated in growth medium plus 1X penicillin/streptomycin with inhibitor for 5 days. A panel of 13 compounds was applied to the cell lines at six different doses in combination with six different doses of a KRAS G12C inhibitor. Cell viability was determined using CellTiter Glo (Promega, Madison, WI) after 5 days of drug treatment. Combination dose points for inhibitors were selected from single-agent dose-response curves of each compound and were defined as the six drug concentrations that showed single-agent inhibition ranging from 0% to 85%. Two biological replicates for each combination matrix per cell line were assessed. Combinations were considered synergistic if the potency of the KRAS G12C inhibitor was increased in the presence of another compound relative to the inhibitor alone.

### Synergy assessment using the Bliss model

The analysis was run in Genedata Screener using viable cell data from CellTiter Glo and normalized as a percentage change from DMSO control wells. Compound identifications and concentrations were applied using a compound mapping table (cmt) file derived from the Echo transfer log file. Dose-response curves were fitted with Genedata Screener using a four-parameter Hill fit with automated outlier detection and all parameters floating with a constraint on maximal effect equal to or greater than −100% activity. Based on these fits, the predicted synergy response for each matrix was calculated according to the Bliss independence model implemented in Genedata Screener Compound Synergy Extension or Synergy Finder 3.0 [20]. Combination matrix analyses and Bliss analyses were performed separately for each of the individual biological replicates and then averaged for an overall Bliss score of the drug combination.

To rank-order compounds by taking their synergism levels into account, we also included the combination index (CI) [21] and excess over Bliss volume. Normalized synergy scores were calculated for each cell line using two different cutoff criteria: (1) a more permissive criterion dividing the synergy score by the median absolute deviation (MAD); and (2) a more stringent criterion dividing the synergy score by the normal approximation of the MAD, the standard deviation approximate (SDapprox). Four cutoff criteria were used to identify synergy hits: > 2 × MAD, > 2 × SDapprox, excess over Bliss volume average >2, and median CI ≤ 0.5.

### Cellular viability assays

Cells were plated in 40-µL growth media containing 1X penicillin/streptomycin at a concentration of 500 cells/well (lung cancer cell lines) or 1000 cells/well (colorectal cancer cell lines). Cells were dispensed directly into 384-well, white-walled, clear-bottomed tissue culture plates containing nanoliter volumes of either single-agent compounds, a 6 × 6 combination matrix for the initial drug screen, or a 10 x 10 combination matrix for hit confirmation. Plates were placed in an incubator at 37°C with 5% CO_2_ for 5 days. On day 5, 40 µL/well of CellTiter Glo reagent was added to the plates. Luminescence readings were obtained using a PHERAstar (BMG Labtech, Cary, NC) after 10 minutes at room temperature. IC_50_ values were calculated using Genedata Screener software. For purposes of hit confirmation, combinations of interest were re-run in a 10 x 10 combination matrix.

### FGFR silencing

Dharmacon siRNA targeting reagents were purchased from Horizon Discovery (Lafayette, CO); both SMARTpool and individual siRNA targeting different sequences of FGFR were assessed. The LU99 cell line was grown and transfected with siRNAs using DharmaFECT 1 reagent (Dharmacon™, Horizon Discovery) according to manufacturer instructions. For Western blot lysates, cells were seeded in six-well plates at a density of 5 × 10^5^ cells in 4 mL of media and lysed after 48 or 72 hours.

For FGFR siRNA studies combined with KRAS G12C inhibition, cells were seeded in 96-well plates at a density of 2000 cells/well in 100 µL of media. After 48 hours of siRNA treatment, KRAS G12C inhibitors were added to plates in an 11-point dose-response curve and incubated at 37°C with 5% CO_2_. Cell viability was assessed using CellTiter Glo reagent either 72 hours or 120 hours from the beginning of KRAS G12C inhibitor treatment, and luminescence was obtained using a PHERAstar. IC_50_ values were calculated using GraphPad Prism software v9.3.1 (GraphPad Software, Boston, MA) using the function log(inhibitor) versus response-variable slope (four-parameter).

### Antibodies and western blotting

Antibodies directed against the following were obtained from Cell Signaling Technology (Danvers, MA) and were used at a concentration of 1:1000 unless otherwise noted: β-actin (1:5000 dilution, #5125), E-cadherin (#3195), ERK (#4695), FGFR1 (#9740S), FGFR4 (#8562S), FRS2a (#94826), glyceraldehyde 3-phosphate dehydrogenase (GAPDH) (1:2000 dilution, #5174S), pFRS2 (1:500 dilution, #3861), pERK (#4370), vimentin (#5741S). Antibodies against FGFR2 (1:1000 dilution, #ab109372) and FGFR3 (1:2000 dilution, #ab133644) were obtained from Abcam (Waltham, MA). Following siRNA or inhibitor treatment, media was aspirated from the plates, and cells were washed in cold phosphate buffered saline (PBS) and lysed using 1X Cell Signaling Technology lysis buffer containing 1X phosphatase and protease inhibitors. Lysates were subjected to SDS-PAGE using 4–12% Bis-Tris gels (Bio-Rad, Hercules, CA), followed by transfer to Trans-Blot Turbo Nitrocellulose Membranes (Bio-Rad). Membranes were immunoblotted with the described antibodies, and membranes were developed using SuperSignal West Femto Maximum Sensitivity Substrate (Thermo Fisher Scientific, Waltham, MA).

LU99 lung cancer cells were routinely grown in RPMI 1640 with 10% FBS at 37˚C with 5% CO_2_. Cells were seeded at 6 × 10^5^ cells/well of a six-well plate and were allowed to attach for 48 hours before treatment. Compounds were added to duplicate cultures and incubated for 24 hours. To facilitate detection by Western blot, one set of plates received 25 ng/mL of recombinant human (rh)FGF (R&D Systems, Minneapolis, MN) for an additional 15 minutes at the end of the treatment to amplify the FGF signaling pathway protein expression. The cultures were washed with cold PBS, lysed on ice with 1X Cell Lysis Buffer (Cell Signaling Technology) containing 1X Halt Protease and Phosphatase Inhibitors. The lysates were centrifuged at 12,700 rpm for 15 minutes at 4˚C to remove debris and the protein concentrations of the lysate supernatant were determined as instructed for the Pierce BCA Protein Assay (Thermo Fisher Scientific). Lysates were prepared with 6 × Laemmli SDS Sample Buffer (reducing) and heated for 6 minutes at 100˚C. Samples were loaded onto Novex WedgeWell 4–12%, Tris-Glycine, 1.0-mm, Mini Protein gels (Invitrogen, Waltham, MA) at 25 µg of cell lysate per lane, followed by transfer to nitrocellulose membranes using the iBlot dry blotting system (Thermo Fisher Scientific). Membranes were immunoblotted with the described antibodies overnight at 4˚C and developed using SuperSignal West Femto Maximum Sensitivity Substrate.

### Resistant cells

MIA PaCa-2 pancreatic cancer cells expressing the KRAS G12C mutation were obtained from ATCC and were maintained in RPMI 1640 with 10% FBS supplemented with increasing concentrations of AMG 510 over time, until the cells were routinely growing in the presence of 1 µM AMG 510. The resistant pool of cells was cloned by limiting dilution in 96-well plates in the presence of 1 µM AMG 510. Several of the AMG 510–resistant clones were characterized by Western blot, with particular interest in markers of EMT transition, such as vimentin.

### In vivo studies

LU99 lung cancer cells were obtained from the Japanese Collection of Research Bioresources Cell Bank and were maintained in RPMI 1640 with 10% FBS. Ten million LU99 cells were inoculated in the left flank of 6- to 8-week-old female NCr nude mice (Taconic Biosciences, Germantown, NY). Cells were re-suspended in 1:1 ratio of PBS and Matrigel (Corning Life Sciences, Tewksbury, MA). PDX studies were performed at Crown Bioscience (San Diego, CA). Pemigatinib and AMG 510 were suspended in 5% N,N-dimethyl acetamide (DMAC) plus 50 mM citrate buffer (pH 3.0) in 0.5% methyl cellulose. MRTX849 was formulated in 10% Captisol (Cydex Pharmaceuticals, Lenexa, KS) in 50 mM citrate buffer (pH 5.0). When tumors were established, female NOD/SCID or BALB/c nude mice were randomized by tumor volume in groups of nine or ten per group (n = 5 per group for PDX studies) before initiation of treatment. All compounds were orally dosed once daily until study end.

For measurement of phospho-ERK (pERK) and ERK levels, mice with established tumors were collected 2 or 6 hours after one dose of single-agent or combination treatment, and pERK/ERK levels were quantified by MSD Phospho/Total ERK1/2 Whole Cell Lysate Assay Kit (Meso Scale Discovery, Rockville, MD). Tumor volume was calculated using the formula (L × W^2^)/ 2, where L and W refer to the length and width dimensions, respectively. Tumor growth inhibition was calculated using the formula (1 − (V_T_/ V_C_)) × 100, where V_T_ is the tumor volume of the treatment group on the last day of treatment, and V_C_ is the tumor volume of the control group on the last day of treatment. Two-way analysis of variance with Dunnett’s multiple comparisons test was used to determine statistical differences between treatment groups (GraphPad Prism). Mice were housed at five to ten animals per cage, were provided enrichment and exposed to 12-hour light/dark cycles, and were monitored daily. The number of mice used per treatment group was based on historical experience with each tumor model. Mice whose tumor volumes exceeded limits (10% of body weight) or had 20% body weight loss that was associated with clinical symptoms were immediately humanely euthanized by CO_2_ inhalation. Of the 244 animals used in this study, no animals died before meeting the criteria for euthanasia. Four animals were euthanized due to body weight loss of >20% in the LU5191 PDX model. Duration of in vivo studies lasted between 12–62 days, depending on the tumor model. Animals were maintained in barrier facilities fully accredited by the Association for Assessment and Accreditation of Laboratory Animal Care International, and all procedures were conducted in accordance with the US Public Service Policy on Human Care and Use of Laboratory Animals and with Incyte’s or Crown Bio’s Animal Care and Use Committee (IACUC) Guidelines. For the humane sacrifice of animals, mice were euthanized using carbon dioxide (CO_2_) inhalation, followed by cervical dislocation to ensure death. Efforts to minimize suffering included careful monitoring of the animals for signs of distress and providing supportive care as needed.

### EMT gene signature analysis

Processed RNA-seq profiling data for PDX models was supplied by Crown Bioscience. Briefly, the quality of RNA-seq raw data were checked by FastQC software (Babraham Bioinformatics, Cambridge, UK) and adapter and low-quality sequences were trimmed using Trimmomatic [22]. The reads were then mapped to human (hg19) and mouse genomes (mm10) with STAR [23], and reads with preferred mapping to the mouse genomes were removed. Remaining reads were mapped to reference genes (ENSEMBL GRCh37.66) using Bowtie [24], and gene expression was calculated by MMSEQ [25]. Normalized expression values (log2 [FPKM + 0.01]) were used as input to singscore [26] using the 77-gene pan-cancer epithelial-mesenchymal signature described by Mak et al. [27]. Genes in this signature are associated with either epithelial or mesenchymal states, with higher resulting scores representing a more mesenchymal phenotype, and lower scores representing a more epithelial phenotype. Pan-cancer RNA-seq FPKM data were obtained from the OncoLand Cancer Dataset Database (QIAGEN, Redwood City, CA) for The Cancer Genome Atlas (TCGA) samples (version Q3 2021). Similar to the PDX sample RNA-seq signature score generation, each TCGA sample was scored using singscore, with the EMT signature genes and normalized expression values (log2 [FPKM + 0.01]) as input.

## Results

### KRAS G12C combination studies

We performed a combination screen to identify agents that demonstrate synergistic effects with KRAS G12C inhibitors. One compound of interest that emerged from this screen was the FGFR1–3 inhibitor, pemigatinib. The combination of AMG 510 with pemigatinib in several KRAS G12C mutated cell lines resulted in high Bliss synergy scores (Table 1).

**Table 1:** Table of Bliss synergy scores in KRAS G12C–mutant cell lines treated with AMG 510 and pemigatinib (FGFR inhibitor).

Compound	G12C inhibitor	Average Bliss scores per cell line							
		H1792	SW1573	Calu-1	HCC-44	H2030	H2122	H358	SW837
Pemigatinib	AMG 510	37	25.9	11.2	5.6	16.3	3.9	3.7	−2.6

FGFR, fibroblast growth factor receptor; G12C, glycine to cysteine substitution of KRAS at amino acid 12.

Hit confirmation of pemigatinib was further assessed in an expanded 10 × 10 drug combination matrix with AMG 510 across KRAS G12C-mutant lung cancer cell lines, including the previously identified mesenchymal-like cell line LU99 [18]. We also included SW837 colon cancer cells that have an epithelial-like phenotype [28]. Bliss scores in this expanded combination matrix with the combination of AMG 510 and pemigatinib were most significant in mesenchymal-like cell lines with minimal synergy observed in epithelial-like cell lines (Table 2).

**Table 2:** Table of Bliss synergy scores in KRAS G12C–mutant cells with an epithelial or mesenchymal phenotype treated with the combination of AMG 510 and pemigatinib.

Cell line	Tissue	Type	Average Bliss score
SW837	CRC	Epithelial	−7.7
H358	Lung	Epithelial	3.7
H2122	Lung	Epithelial	3.9
H2030	Lung	Mesenchymal	3.4
HCC-44	Lung	Mesenchymal	4.4
Calu-1	Lung	Mesenchymal	11.2
SW1573	Lung	Mesenchymal	25.9
H1792	Lung	Mesenchymal	26.7
LU99	Lung	Mesenchymal	41.8

CRC, colorectal cancer; G12C, glycine to cysteine substitution of KRAS at amino acid 12. Classification of cell lines as mesenchymal or epithelial was based on published literature [18,29].

### FGFR1 mediates acquired drug resistance

**Fig 1:**
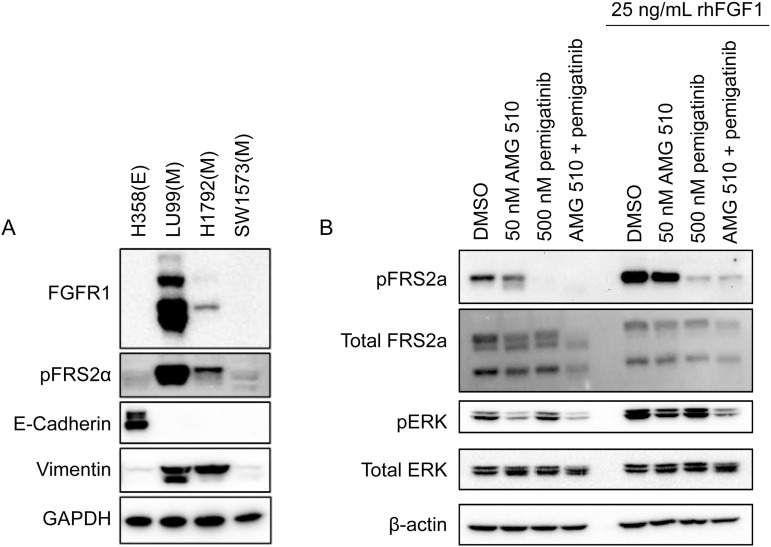
Cell line characterization and combination effect of KRAS G12C inhibitors and pemigatinib. (A) Western blot comparison of cell lines H358, LU99, H1792, and SW1573 (lanes 1–4). Cell lysates were compared for FGFR1, pFRS2α, E-cadherin, and vimentin as markers of epithelial (E) or mesenchymal (M) cell phenotype. (B) LU99 cells were treated with 50 nM AMG 510, 500 nM pemigatinib, or the combination for 24 hours. rhFGF was added at 25 ng/mL for 15 minutes before harvesting to amplify the pFRS2α expression to a level detectable by Western blot. Loading controls were assessed by Western blotting for β-actin. DMSO, dimethyl sulfoxide; ERK, extracellular signal-regulated kinase; FGF, fibroblast growth factor; FGFR, fibroblast growth factor receptor; FRS, fibroblast growth factor receptor substrate; i, inhibitor; p, phospho; rh, recombinant human.

Markers of mesenchymal and epithelial phenotypes, as well as FGFR1 expression, were assessed in three mesenchymal cell lines that exhibited high synergy scores with the KRAS G12C inhibitor plus pemigatinib combination, and in one epithelial cell line that did not exhibit synergy (H358) (Fig 1A). Notably, LU99, which exhibited the highest synergy score, expressed the highest levels of FGFR1. Phosphorylation of the FGFR adaptor protein FRS2, a biomarker of FGFR pathway activation, was also highest in LU99 cells, although it was faintly detected in H1792 cells. LU99 and H1792 expressed the mesenchymal marker vimentin, whereas H358 cells expressed the epithelial marker E-cadherin.

Mesenchymal-like LU99 cells were treated with AMG 510, pemigatinib, or the combination (Fig 1B) and levels of pFRS2 and pERK were assessed. Cells were treated with or without 25 ng/mL recombinant FGF following 24 hours of treatment with test compounds. Treatment of FGFR1-high KRAS G12C–mutant LU99 cells with AMG 510 did not result in increased FRS2 phosphorylation, however pERK was partially inhibited. Pemigatinib treatment also did not alter pERK levels. In contrast, the combination of AMG 510 and pemigatinib reduced the levels of pERK greater than AMG 510 or pemigatinib alone. Both pemigatinib and the combination decreased pFRS2 to similar levels. These data demonstrate that pemigatinib combined with AMG 510 resulted in a cooperative effect in the inhibition of pERK signaling.

**Fig 2:**
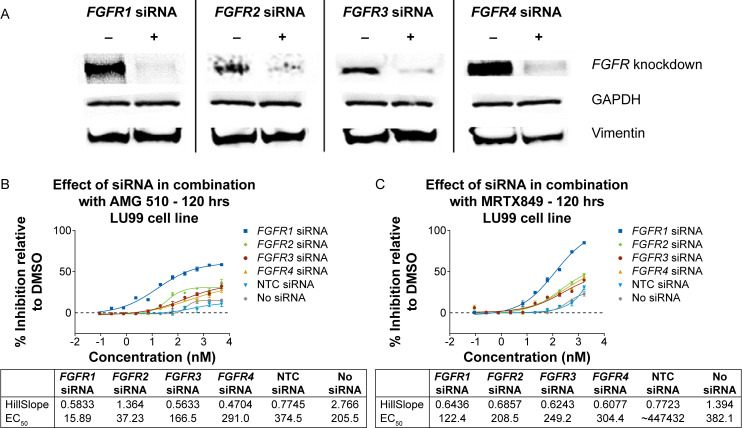
*FGFR1* siRNA knockdown synergizes with KRAS G12C inhibitors in LU99 cells. (A) siRNA knockdown of *FGFR1-4* in LU99 mesenchymal-like cell line. Western blot indicates successful siRNA knockdown of *FGFR1-4* in LU99 cells. Vimentin was used as a marker to indicate mesenchymal phenotype. LU99 cells were treated with individual siRNA for 48 hours before lysis and immunoblotting. LU99 mesenchymal-like cells were treated with individual siRNA for *FGFR1-4* for 48 hours followed by (B) AMG 510 or (C) MRTX849 treatment for 120 hours. *FGFR1* knockdown combined with KRAS G12C inhibition demonstrates increased drug sensitivity. DMSO, dimethyl sulfoxide; EC_50_, half-maximal effective concentration; FGFR, fibroblast growth factor receptor; G12C, glycine to cysteine substitution of KRAS at amino acid 12; GAPDH, glyceraldehyde 3-phosphate dehydrogenase; NTC, non-targeting control; siRNA, small interfering RNA.

To confirm the contribution of the *FGFR1–4* genes in feedback activation following KRAS G12C inhibition, functional siRNA knockdown experiments were performed, where each individual FGFR gene was silenced. Silencing of the four FGFR genes was confirmed by Western blot in LU99 cells (Fig 2A). When LU99 cells were treated with AMG 510 (Fig 2B) or MRTX849 (Fig 2C), inhibition of FGFR1 resulted in the highest level of synergistic activity. In contrast, knockdown of the FGFR2, 3, or 4 genes showed a markedly less significant effect of combination treatment. These data identified FGFR1 as the primary mediator of feedback activation following KRAS G12C inhibition.

**Fig 3:**
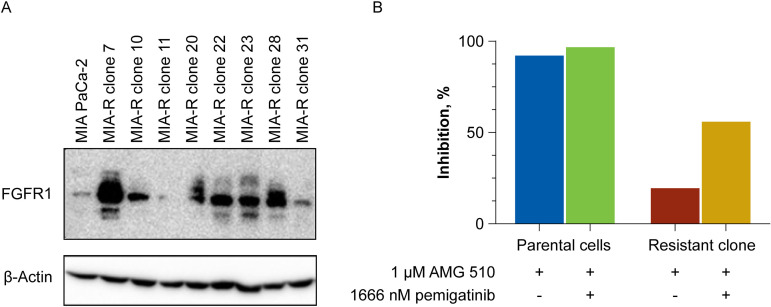
FGFR1 expression and combination effect with AMG 510 and pemigatinib in MIA PaCa-2–resistant cells. (A) Western blot showing elevated FGFR1 expression in MIA PaCa-2 clones resistant to 1 µM AMG 510. (B) MIA PaCa-2 parental cells and resistant clone 7 were treated with 1 μM AMG 510 alone or in combination with pemigatinib for 5 days. Percent growth inhibition is shown. FGFR, fibroblast growth factor receptor.

To determine whether increased FGFR1 activity may be an acquired resistance mechanism, MIA PaCa-2 cells resistant to AMG 510 were generated. MIA PaCa-2 was chosen to create AMG 510-resistant clones, as this cell line has been characterized as epithelial-like and previously utilized in resistance-generation models. Clones were derived once the cells were able to grow in the presence of 1 µM AMG 510. Subsequent protein analysis identified high levels of FGFR1 expression in a subset of resistant clones (Fig 3A). MIA PaCa-2–resistant clones and parental cells were then evaluated side by side in cell viability assays investigating the effect of AMG 510 compared with AMG 510 combined with pemigatinib (Fig 3B). The combination of AMG 510 plus pemigatinib in MIA PaCa-2–resistant clone 7 inhibited cell proliferation to a greater extent than AMG 510 alone. The combination of pemigatinib and AMG 510 resulted in higher Bliss scores when compared with the combination of an FGFR2-specific inhibitor, RLY-4008 [30] and AMG 510, supporting the siRNA data that FGFR2 was not responsible for feedback inhibition following KRAS G12C inhibitor treatment (Table 3).

**Table 3:** Table of MIA PaCa-2–resistant clones and Bliss synergy scores from the combination of AMG 510 and pemigatinib or AMG 510 and an FGFR2/3i.

Cell line	AMG 510 + pemigatinib	AMG 510 + RLY-4008
MIA PaCa-2	6.3	7.5
MIA PaCa-2 clone 7	15	6.7
MIA PaCa-2 clone 11	24.9	9.3
MIA PaCa-2 clone 23	16.1	7.5
MIA PaCa-2 clone 28	21.8	˗2.6

FGFR, fibroblast growth factor receptor; i, inhibitor.

### In vivo effects of KRAS G12C inhibition in combination with pemigatinib

**Fig 4:**
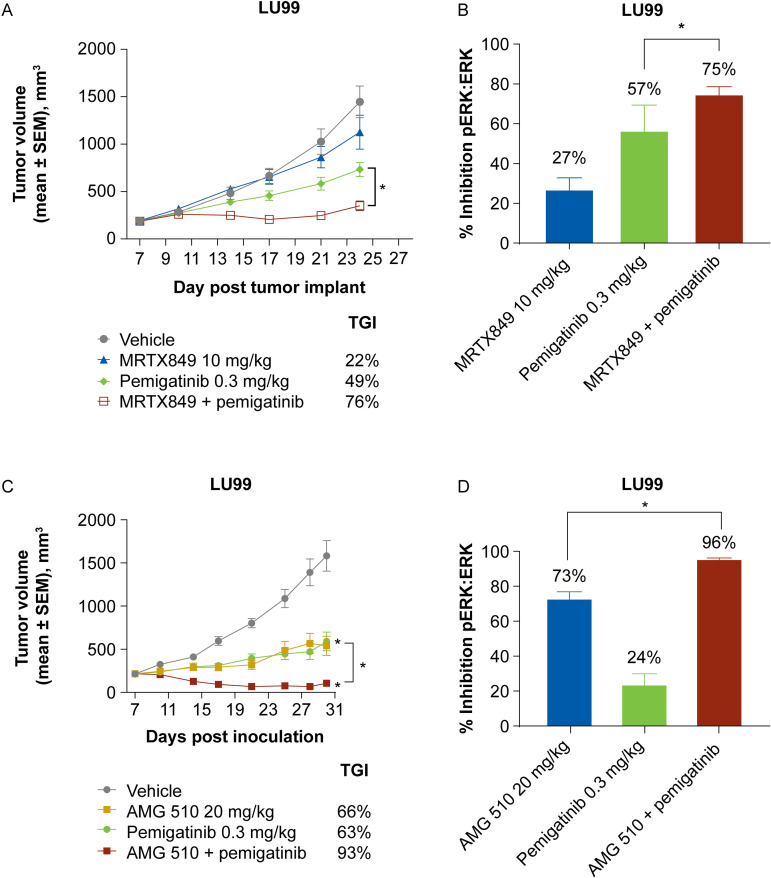
In vivo activity of KRAS G12C inhibitors and pemigatinib in the LU99 xenograft model. (A) Nude mice bearing LU99 tumors were dosed orally with MRTX849, pemigatinib, or the combination at the indicated doses starting on day 7 post tumor implant. (B) LU99 tumor–bearing nude mice (tumor volume ~390 mm^3^) were given a single dose of the indicated compounds, and tumors were harvested 6 hours post dose. The ratio of pERK/ERK was measured using a Phospho/Total ERK1/2 Whole Cell Lysate Assay Kit (Meso Scale Discovery). (C) Nude mice bearing LU99 tumors were dosed orally with AMG 510, pemigatinib, or the combination at the indicated doses starting on day 7 post tumor implant. (D) LU99 tumor–bearing nude mice (tumor volume ~328 mm^3^) were given a single dose of the indicated compounds, and tumors were harvested 2 hours post dose. The ratio of pERK/ERK was measured using a Phospho/Total ERK1/2 Whole Cell Lysate Assay Kit. *P < 0.05, based on one-way ANOVA. ANOVA, analysis of variance; ERK, extracellular signal-regulated kinase; p, phospho; SEM, standard error of the mean; TGI, tumor growth inhibition.

A role for FGFR-mediated resistance was further supported with in vivo studies of KRAS G12C–mutant xenografts. Consistent with in vitro studies, in the LU99 xenograft model, the combination of MRTX849 and pemigatinib resulted in increased anti-tumor activity compared with either agent alone (Fig 4A). Examination of tumors harvested at the end of study showed an increase in the inhibition of pERK/ERK ratios in the combination group compared with single-agent treatment (Fig 4B). A similar combination effect and increased inhibition of pERK were observed with the combination of AMG 510 and pemigatinib (Fig 4C–D).

To further explore the activity of KRAS G12C inhibitor plus pemigatinib combinations, we applied an EMT signature analysis to available gene expression data for a set of NSCLC PDX models in order to select models with more mesenchymal or epithelial phenotypes [27] (S1 Fig). The PDX models were classified as mesenchymal if the EMT score was greater than or equal to a pan-cancer median score (−0.05), determined by applying the signature to available RNA-seq expression data from TCGA samples (S2 Fig; S1 Table). Seven NSCLC PDX models were selected that possessed mesenchymal- or epithelial-like gene signatures. Western blot analysis confirmed that the four PDX models classified as mesenchymal-like expressed FGFR1 (LU11722, LU5200, LU11612, and LU5191) (S3 Fig). Low levels of FGFR1 were also detected in two of the three models classified as epithelial-like (LU6405 and LU5245). FGFR1 protein expression was absent in a third epithelial-like model LU9359 (S3 Fig), which was consistent with the absence of *FGFR1* messenger RNA expression in this model (S1 Table). The vimentin protein expression observed in the epithelial-like models is likely due to the expression of vimentin in stromal cells present in the tumor.

**Fig 5:**
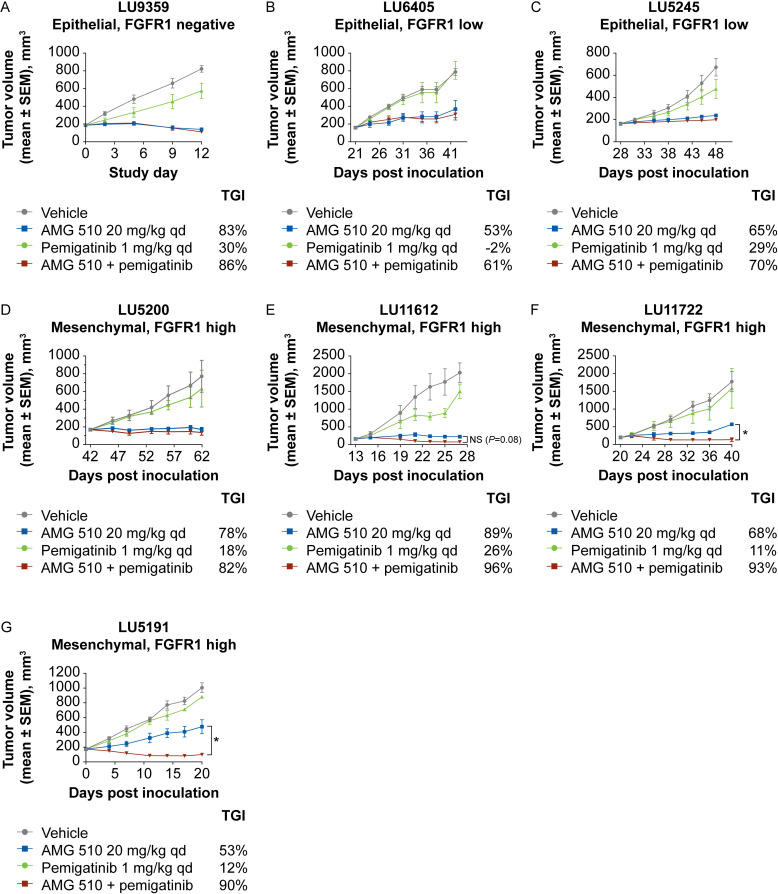
In vivo combination activity of AMG 510 and pemigatinib in epithelial-like or mesenchymal-like phenotype lung cancer PDX models. Immunodeficient mice bearing NSCLC PDX tumors were orally dosed with AMG 510 (20 mg/kg), pemigatinib (1 mg/kg), or the combination once daily. (A) LU9359, (B) LU6405, and (C) LU5245 epithelial-like phenotype; (D) LU5200, (E) LU11612, (F) LU11722, and (G) LU5191 mesenchymal-like phenotype. *P < 0.05, based on two-way ANOVA with Dunnett’s multiple comparisons test. ANOVA, analysis of variance; FGFR, fibroblast growth factor receptor; NS, not statistically significant; NSCLC, non-small cell lung cancer; PDX, patient-derived xenograft; qd, once daily; SEM, standard error of the mean; TGI, tumor growth inhibition.

The combination of AMG 510 and pemigatinib was evaluated in these PDX models in vivo. No increase in combination activity was observed in the LU9359 epithelial-like model lacking FGFR1 expression (Fig 5A), or in the LU6405 and LU5245 epithelial-like models expressing low levels of FGFR1 (Fig 5B–C). In contrast, significant combination benefit was observed in the LU11722 and LU5191 models with a mesenchymal phenotype and expressing FGFR1, and a non-significant combination benefit was observed in the LU11612 model (Fig 5D–G). These data support the hypothesis that mesenchymal NSCLC tumors expressing FGFR1 may be preferentially sensitive to the combination of KRAS G12C and FGFR1 inhibitors.

## Discussion

Nearly 40 years after the discovery of KRAS as an oncogene, two small molecule drugs, sotorasib (AMG 510) and adagrasib (MRTX849), were approved by the FDA for KRAS G12C–mutated NSCLC in 2021 and 2022 [9], respectively. Both agents retain KRAS G12C in its GDP-bound inactive state by covalently binding to the mutated cysteine residue within the switch II region, and targeting KRAS G12C led to inhibition of downstream MAPK and PI3K signaling, evidenced by diminished phosphorylation of MEK, ERK, and S6 [5,8]. Although developing KRAS mutation–selective inhibitors has demonstrated efficacious clinical responses, a significant percentage of patients do not respond well to KRAS G12C inhibitors; furthermore, patients who initially experience clinical responses eventually develop acquired resistance to KRAS G12C inhibitors [3,12,13].

Understanding resistance mechanisms of KRAS G12C inhibitors and exploration of potential combination strategies remain major areas of research. [17,18,31–38] A combination screen was performed to identify compounds that have synergistic effects with KRAS G12C inhibitors. In mesenchymal-like cell lines, we identified a clear synergistic effect when combining KRAS G12C inhibitors and pemigatinib suggesting that FGFR activation is a key survival pathway for mesenchymal-like cancer cells treated with KRAS G12C inhibitors. Treatment of mesenchymal lung cancer LU99 cells with AMG 510 and pemigatinib resulted in a significant decrease in pERK levels compared to single-agent treatment, supporting the increased anti-proliferative activity using these two agents. Using genetic tools, we showed that *FGFR1* is the major gene involved in pathway activation, and not *FGFR2*, *FGFR3*, or *FGFR4*.

Aberrant activation of ligand-dependent or ligand-independent FGF/FGFR signaling has been shown to contribute to resistance by promoting proliferation, survival, angiogenesis, and EMT [17,18,34–37]. Our findings support previous studies conducted by Kitai et al. [18] and Manchado et al. [38] demonstrating that in pancreatic and lung cancer cells with mesenchymal phenotypes, inhibition of the MAPK/ERK pathway, downstream of KRAS, results in feedback activation of FGFR1 through downregulation of Sprouty protein expression and resultant activation of pFRS2; the increase in FGFR pathway signaling promotes adaptive resistance. Notably, Kitai et al. reported that in epithelial-like KRAS mutant lung cancer cell lines, feedback activation is mainly driven by the EGFR pathway, whereas in mesenchymal-like KRAS mutant lung cancer cell lines, the FGFR1-FRS2 pathway plays a crucial role in the feedback reactivation of MAPK and the upregulation of AKT signaling.

After we established mesenchymal-like and high FGFR1 expression as major features for intrinsic resistance to KRAS G12C blockade, we further explored the cause of acquired resistance to KRAS G12C inhibitors. Through long-term culture of MIA PaCa-2 cells under high concentrations of AMG 510 (sotorasib), resistant clones were generated and characterized. Strikingly, all AMG 510–resistant clones displayed elevated FGFR1 expression. Therefore, treatment of mesenchymal-like KRAS G12C–mutant cells with KRAS G12C inhibitors induced feedback activation of FGFR1 signaling, which results in acquired drug resistance. Furthermore, the combination of pemigatinib with AMG 510 in one of these clones restored response to AMG 510 in vitro, suggesting that the combination may have beneficial effects in patients with acquired resistance to KRAS G12C inhibitors. Our findings in MIA PaCa-2 are consistent with the work by Manchado [38] in pancreatic and mesenchymal lung cancer cells, and appear to reflect a common mechanism of adaptive feedback activation of the FGFR pathway.

In *in vivo* models, we demonstrated that KRAS G12C inhibitors in combination with the FGFR inhibitor pemigatinib was most efficacious compared to single-agent treatment in the LU99 mesenchymal lung cancer model, which was correlated to increased inhibition of pERK in the combination group. In PDX models, the combination of AMG 510 and pemigatinib was also efficacious in 2 of the 4 mesenchymal lung cancer models and trended towards increased activity in another. In contrast, no combination benefit was observed with the combination in epithelial lung cancer PDX models. FGFR1 expression was typically elevated in mesenchymal lung cancer PDX models, and FGFR1 is a marker of EMT transition. However, it is unclear from our current studies the extent to which FGFR1 overexpression directly impacts the pathway activation relative to the impact of the SPRY-pFRS2 feedback mechanism in mesenchymal cells, posited by Kitai et al. While we did see a synergistic effect with the combination of KRAS G12C inhibitors and pemigatinib, we did not observe increased levels of pFRS2 in the highly mesenchymal LU99 cell line. We hypothesize that this may be due to the already high basal levels of pFRS2 in this cell line.

In summary, our results suggest that the combination of G12C inhibitors with FGFR inhibitors such as pemigatinib may improve clinical outcomes in patients with mesenchymal-like lung cancer and KRAS G12C. Additional research is warranted to determine the applicability of these findings in other tumors and mutation types.

## Supporting information

S1 Table Table of PDX models and associated FGFR1 mRNA and protein expression, EMT score, and classified phenotype. (DOCX)

S1 Fig Z-score–transformed gene expression data for EMT signature genes (Mak MP, et al. Clin Cancer Res. 2016;22: 609–620). doi: 10.1158/1078-0432.CCR-15-0876) and FGFR1 across KRAS G12C–positive NSCLC PDX models. Each signature gene row label is brown or blue, indicating expression associated with mesenchymal (M) or epithelial (E) states, respectively. Columns representing PDX models are ordered from lowest (more E) to highest (more M) singscore-derived EMT score and rows are arranged by unsupervised hierarchical clustering of the expression data. EMT, epithelial-mesenchymal transition; FGFR, fibroblast growth factor receptor; G12C, glycine to cysteine substitution of KRAS at amino acid 12; NSCLC, non-small cell lung cancer; PDX, patient-derived xenograft.
(TIF)

S2 Fig Distributions of singscore-based EMT scores for TCGA samples across 32 tumor types using signature genes described in Mak MP, et al. (Clin Cancer Res. 2016;22: 609–620). doi: 10.1158/1078-0432.CCR-15-0876). The pan-cancer median EMT signature score is represented by the dotted line and tumor types are ordered by increasing median signature score. EMT, epithelial-mesenchymal transition; E, epithelial; M, mesenchymal; TCGA, The Cancer Genome Atlas.
(TIF)

S3 Fig Tumors from vehicle-treated mice were collected at the end of study, homogenized, and assessed for expression of FGFR1, vimentin, and β-actin. Cell lysate from LU99 cells was included as a positive control. FGFR, fibroblast growth factor receptor; PDX, patient-derived xenograft.
(TIF)

S1 Raw Images Raw images for western blots and gels. (PDF)

S1 Raw Figures Raw GraphPad Prism graphs with underlying values used for plotted data and measures reported. (PPTX)
