# Supplementary material for: The FGFR inhibitor pemigatinib overcomes cancer drug resistance to KRAS G12C inhibitors in mesenchymal lung cancer
Source: PLoS One. 2025 Aug 11;20(8):e0327588. doi: 10.1371/journal.pone.0327588 (PMC12338787; doi:10.1371/journal.pone.0327588)
Supplement: S1 Table — (DOCX) [file pone.0327588.s001.docx]

**S1 Table.** Table of PDX models and associated FGFR1 mRNA and protein expression, EMT score, and classified phenotype.

| **Model** | ***FGFR1* mRNA  expression (log2)** | **FGFR1  protein expression** | **EMT score** | **Phenotype** |
| --- | --- | --- | --- | --- |
| LU11722P0 | 7.05 | Yes | 0.23 | Mesenchymal |
| LU5200P1 | 6.72 | Yes | 0.01 | Mesenchymal |
| LU11612P1 | 6.06 | Yes | -0.07 | Mesenchymal |
| LU5191P1 | 4.51 | Yes | -0.07 | Mesenchymal |
| LU9359P3 | 0.11 | No | -0.44 | Epithelial |
| LU5245P4 | 5.59 | Low | -0.53 | Epithelial |
| LU6405P4 | 4.49 | Low | -0.57 | Epithelial |

EMT, epithelial-mesenchymal transition; FGFR, fibroblast growth factor receptor; mRNA, messenger RNA; PDX, patient-derived xenograft.
