# Supplementary figures and images for: The FGFR inhibitor pemigatinib overcomes cancer drug resistance to KRAS G12C inhibitors in mesenchymal lung cancer
Source: PLoS One. 2025 Aug 11;20(8):e0327588. doi: 10.1371/journal.pone.0327588 (PMC12338787; doi:10.1371/journal.pone.0327588)

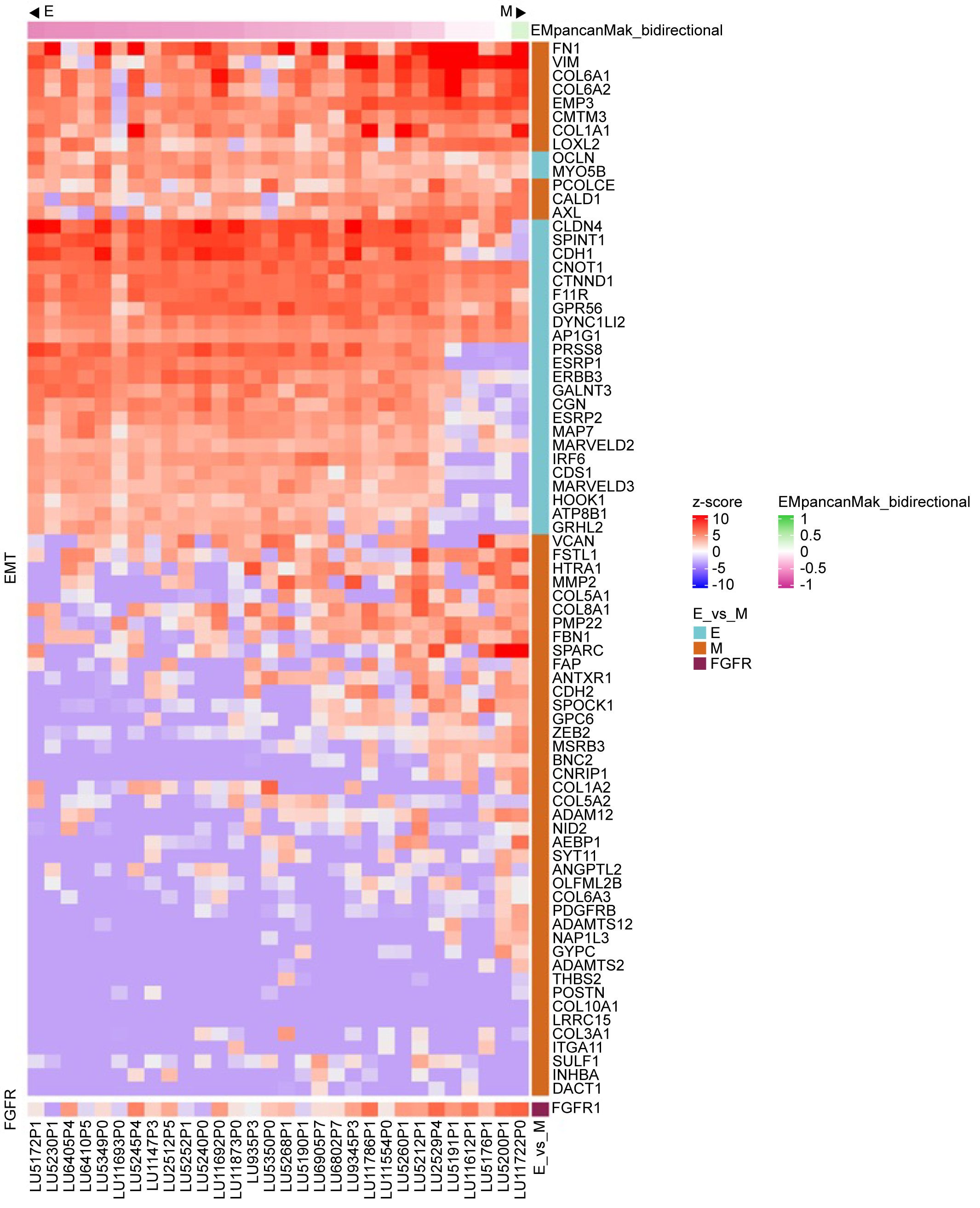

Supplement: S1 Fig — doi: 10.1158/1078-0432.CCR-15-0876) and FGFR1 across KRAS G12C–positive NSCLC PDX models. Each signature gene row label is brown or blue, indicating expression associated with mesenchymal (M) or epithelial (E) states, respectively. Columns representing PDX models are ordered from lowest (more E) to highest (more M) singscore-derived EMT score and rows are arranged by unsupervised hierarchical clustering of the expression data. EMT, epithelial-mesenchymal transition; FGFR, fibroblast growth factor receptor; G12C, glycine to cysteine substitution of KRAS at amino acid 12; NSCLC, non-small cell lung cancer; PDX, patient-derived xenograft. (TIF) [file pone.0327588.s002.tif]

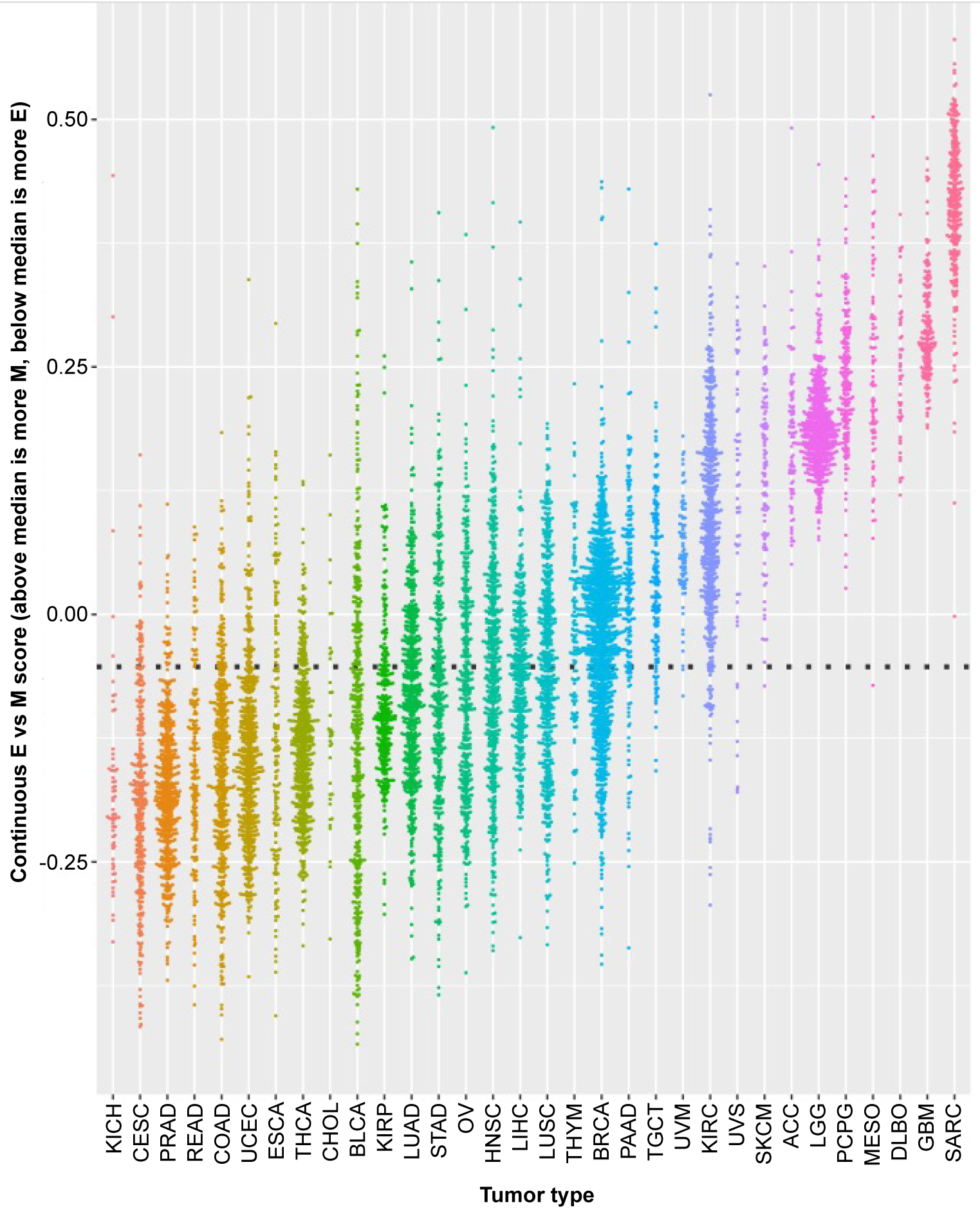

Supplement: S2 Fig — doi: 10.1158/1078-0432.CCR-15-0876). The pan-cancer median EMT signature score is represented by the dotted line and tumor types are ordered by increasing median signature score. EMT, epithelial-mesenchymal transition; E, epithelial; M, mesenchymal; TCGA, The Cancer Genome Atlas. (TIF) [file pone.0327588.s003.tif]

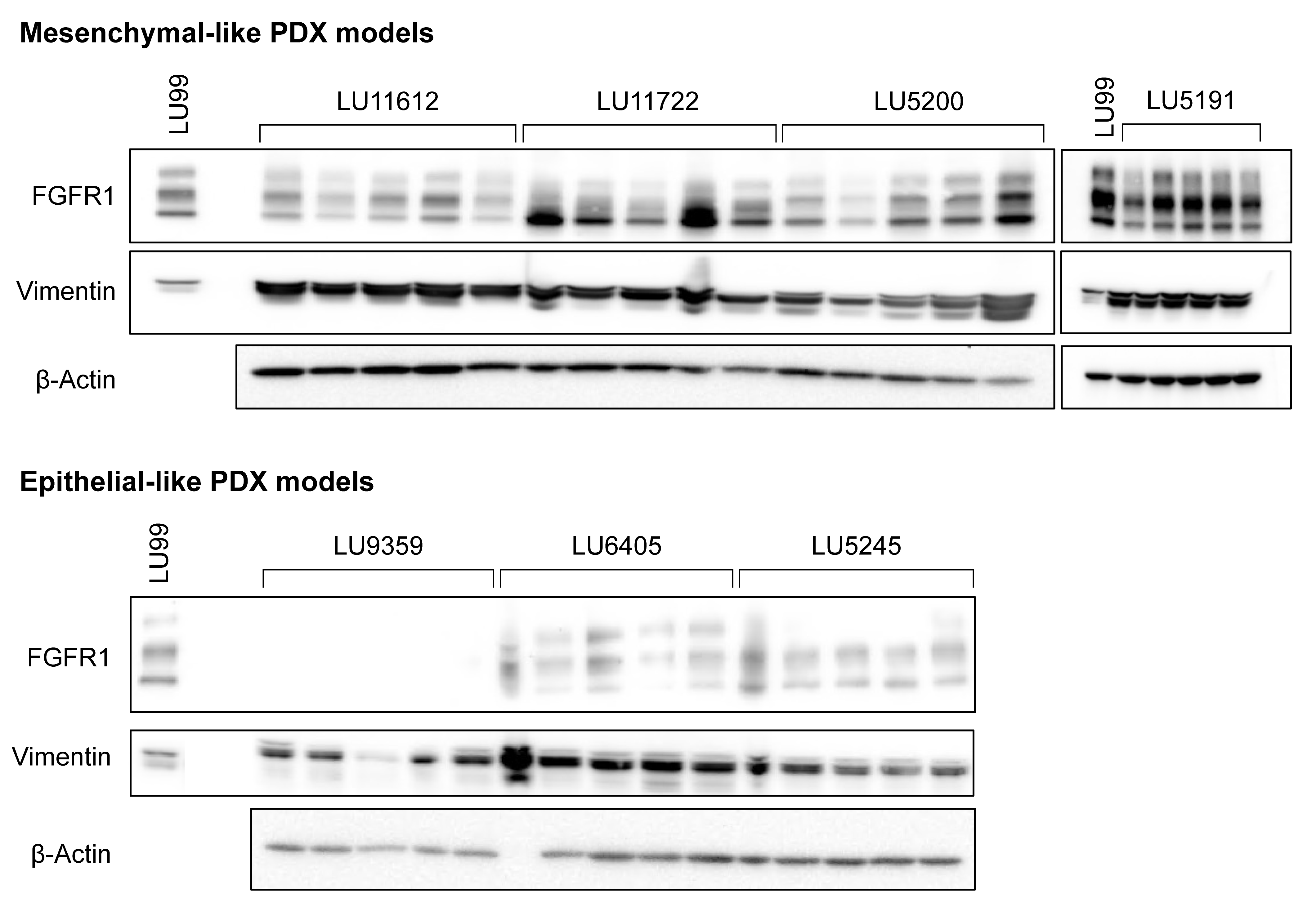

Supplement: S3 Fig — Cell lysate from LU99 cells was included as a positive control. FGFR, fibroblast growth factor receptor; PDX, patient-derived xenograft. (TIF) [file pone.0327588.s004.tif]
