# Supplementary material for: The FGFR inhibitor pemigatinib overcomes cancer drug resistance to KRAS G12C inhibitors in mesenchymal lung cancer
Source: PLoS One. 2025 Aug 11;20(8):e0327588. doi: 10.1371/journal.pone.0327588 (PMC12338787; doi:10.1371/journal.pone.0327588)

The FGFR inhibitor pemigatinib overcomes cancer drug resistance to KRAS G12C inhibitors in mesenchymal lung cancer

Angela Abdollahi, Margaret Favata, Michael Weber, Valerie Roman, Kayla Hammond, Rodrigo Hess, Matthew R. Farren, Mike Schaffer, Aidan Gilmartin, Hui Wang, Jonathan Rios-Doria*, Alejandro Amador-Arjona

Incyte Research Institute, Wilmington, Delaware, United States of America

*Corresponding author: Email: jdoria@incyte.com

S1 Raw Images. Raw Western blots with molecular markers and labeled lanes for reported data.

Figure 1A: Raw and uncropped blots for FGFR1, pFRS2α, E-Cadherin, vimentin and GAPDH

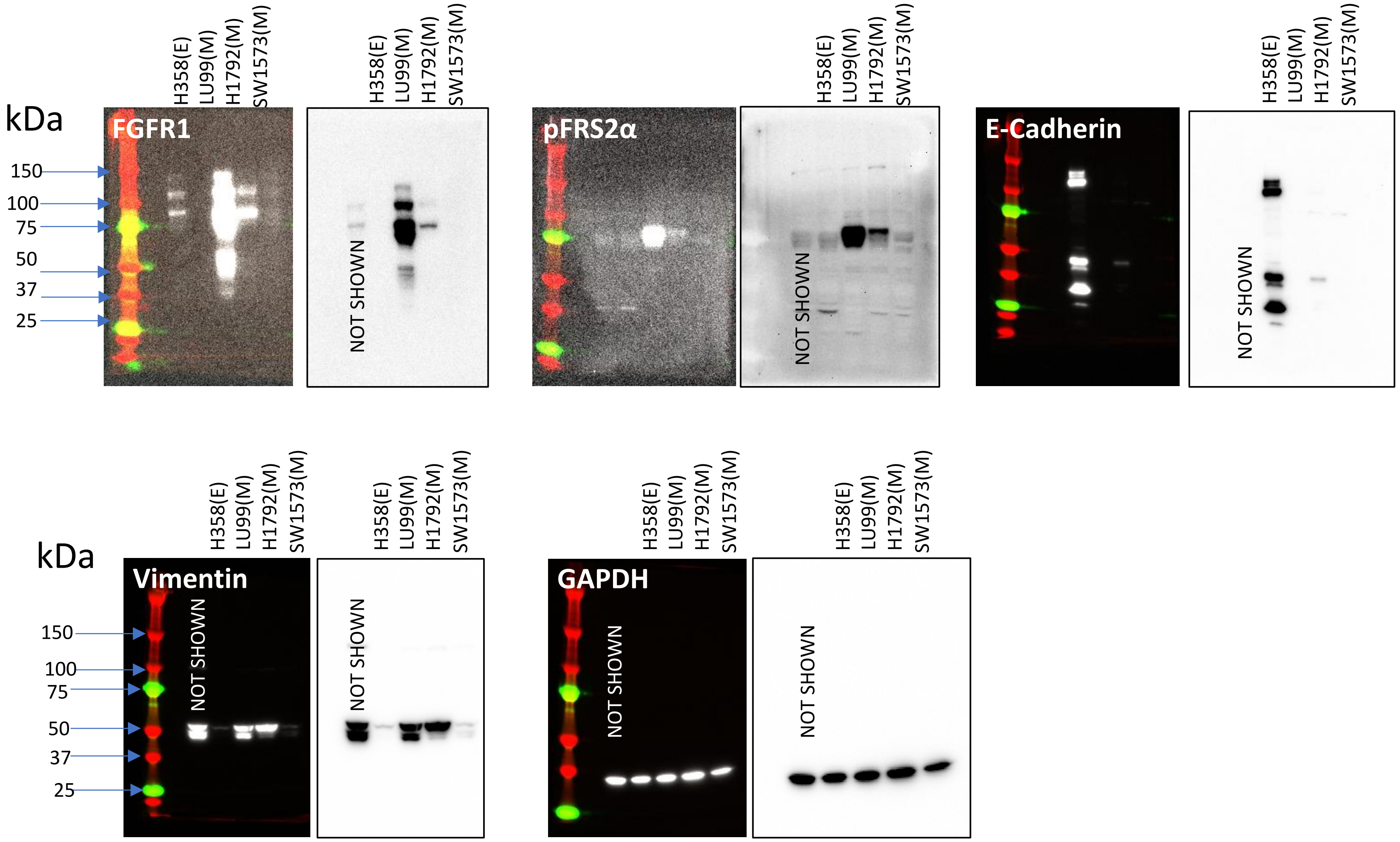

Figure 1B: Raw and uncropped blots for phospho-FRS2α and total FRS2α

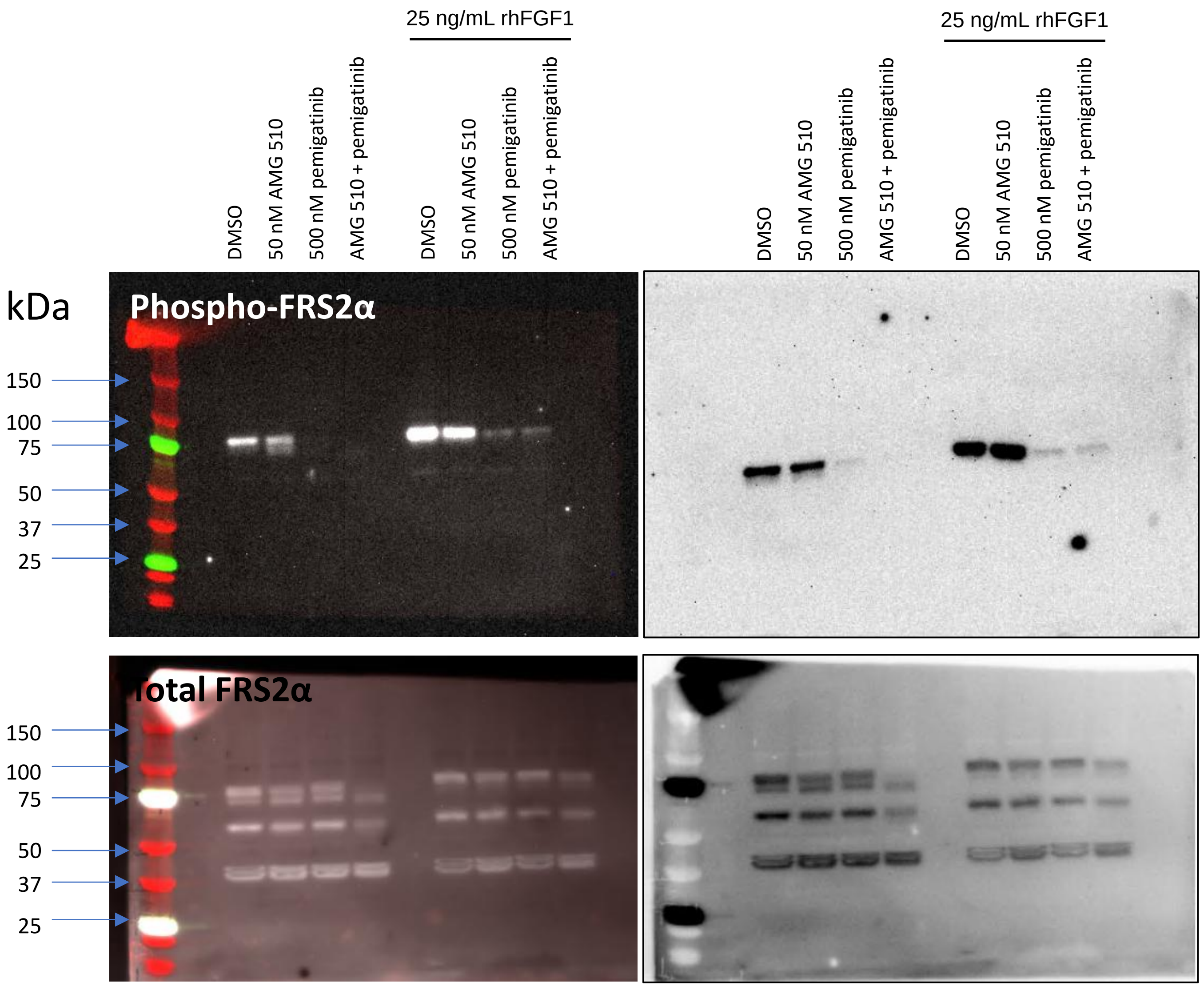

Cell signaling reports 78, 80 and 85 kDa bands in their antibody brochure. Lu99 resembles the bands with RAMOS cells in their brochure. The lower band above is residual Total ERK from the first round of blots around 42 kDa. This blot was stripped and reprobed for total FRS2a but total ERK was still detected as well as FRS2.

Figure 1B: Raw and uncropped Western blots for phospho-ERK and total ERK (Erk1/2)

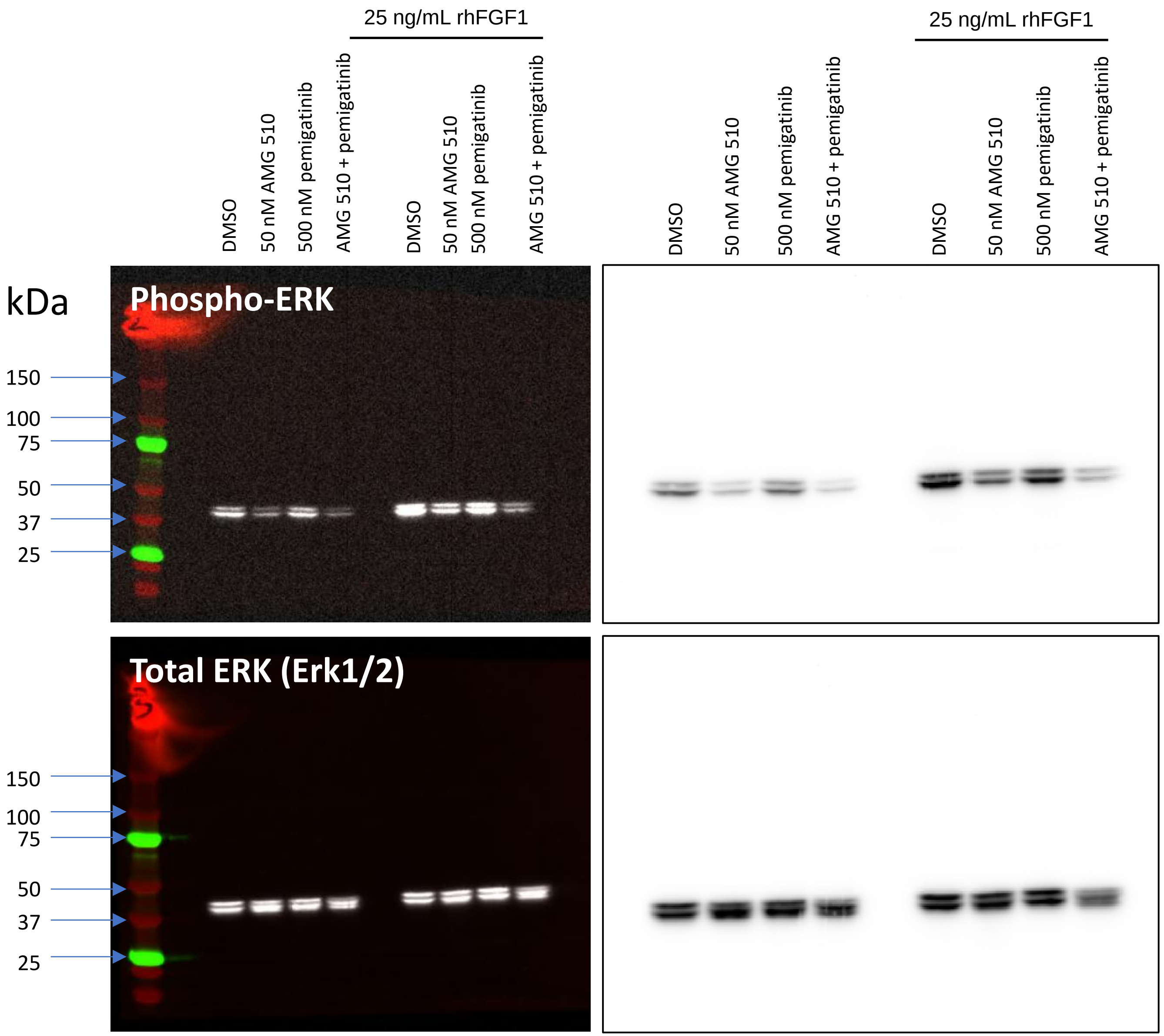

Figure 1B: Raw and uncropped blots for β-Actin-HRP

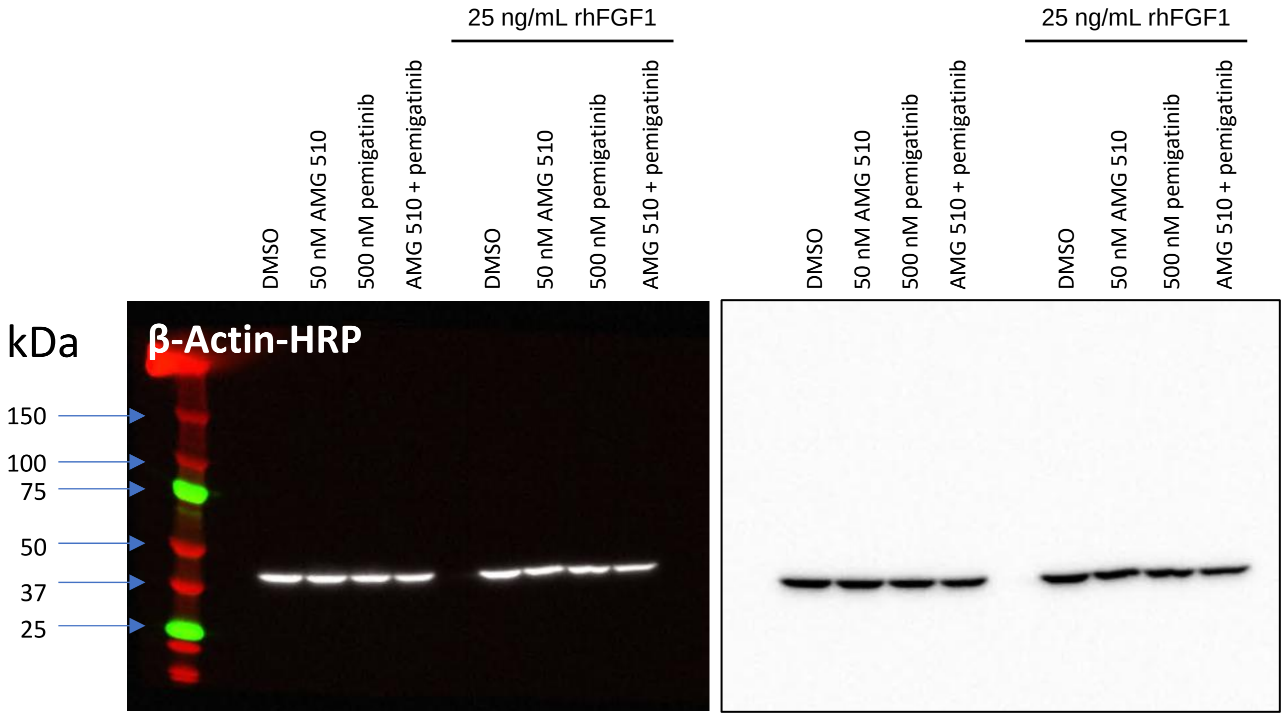

Figure 2A: FGFR1 siRNA raw labelled blots

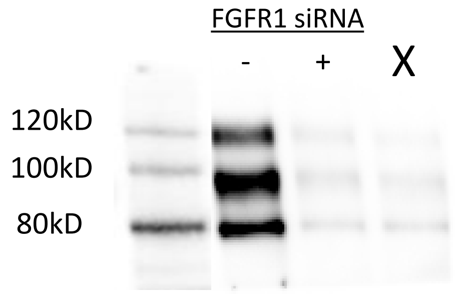

Figure 2A: FGFR2 siRNA raw labeled blots

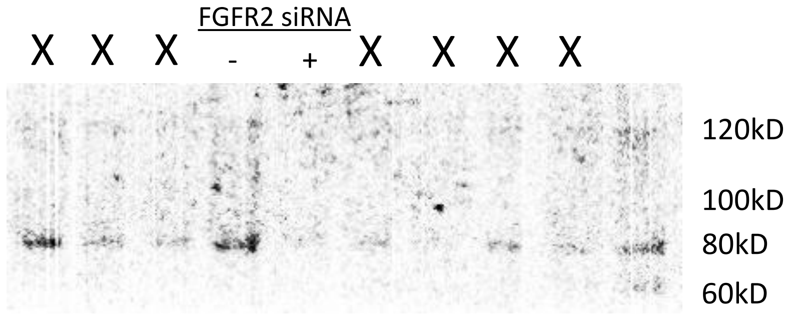

Figure 2A: FGFR3 siRNA raw labeled blots

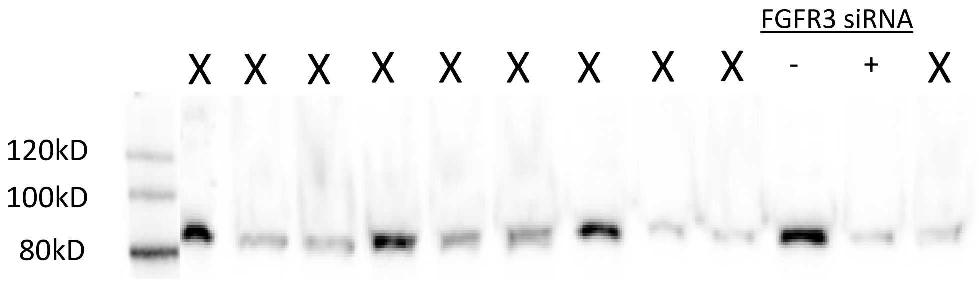

Figure 2A: FGFR4 siRNA raw labeled blots

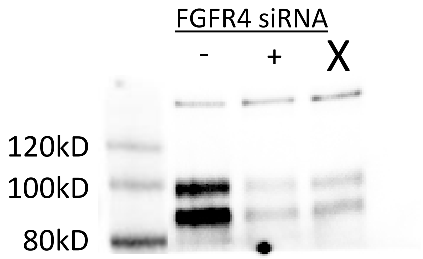

Figure 2A: Vimentin raw labeled blots for FGFR1, FGFR2, FGFR3 and FGFR4 siRNA

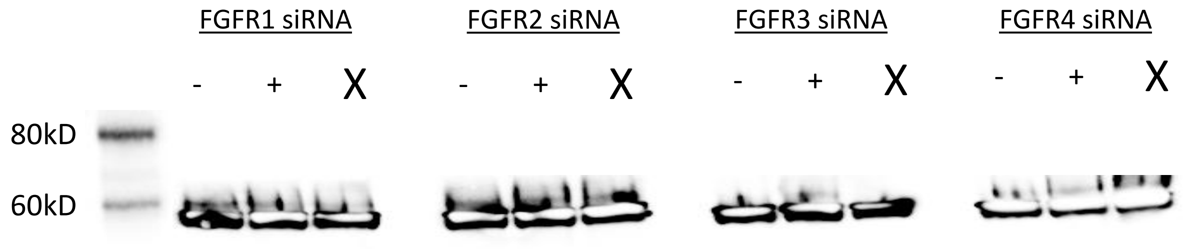

Figure 2A: GAPDH raw labeled blots for FGFR1, FGFR2, FGFR3 and FGFR4 siRNA

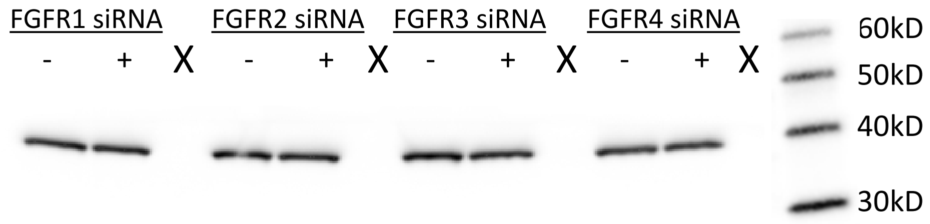

Figure 3A: Raw labeled blots for FGFR1 and β-Actin

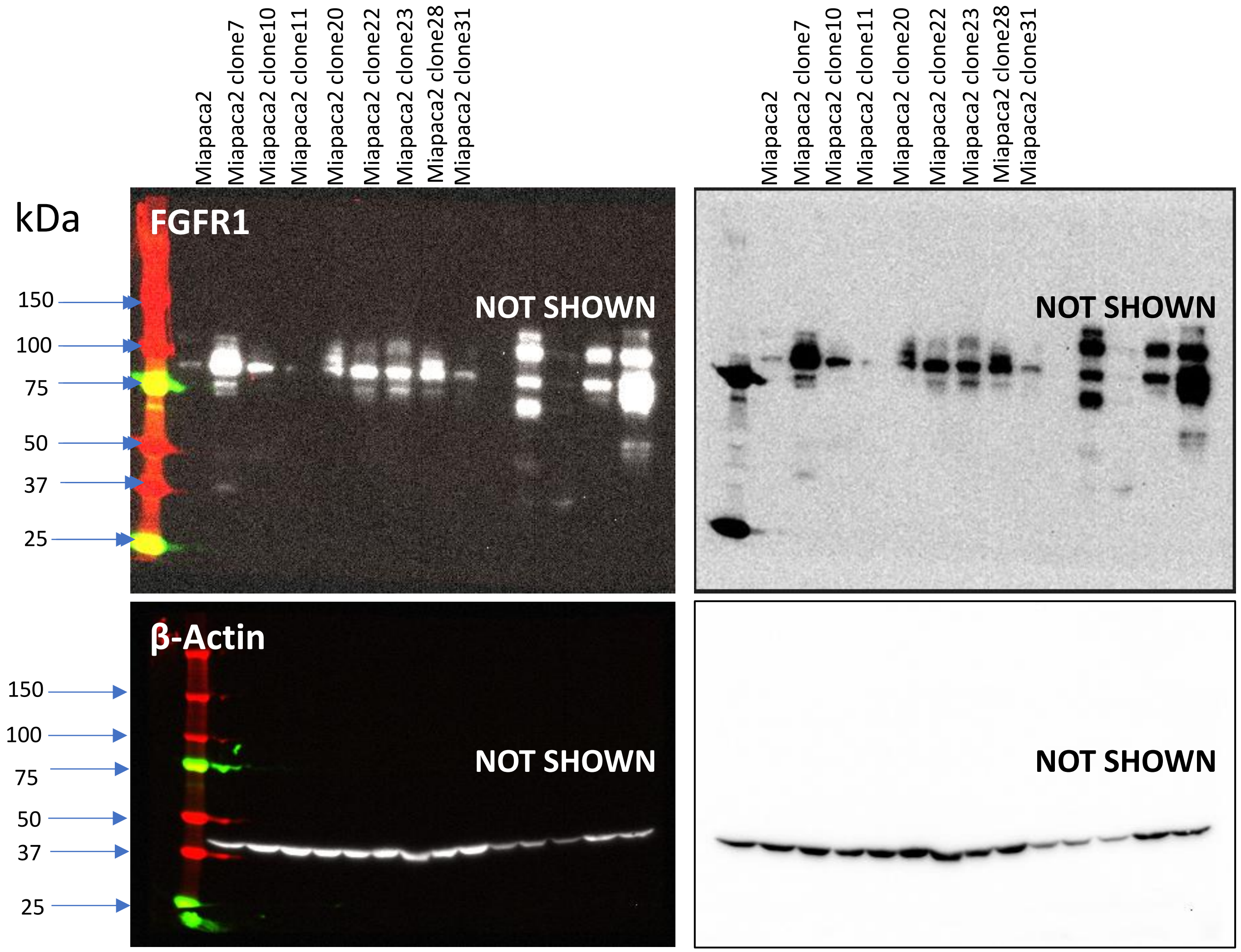

Figure S3: Raw labeled blots for FGFR1, vimentin and β-Actin

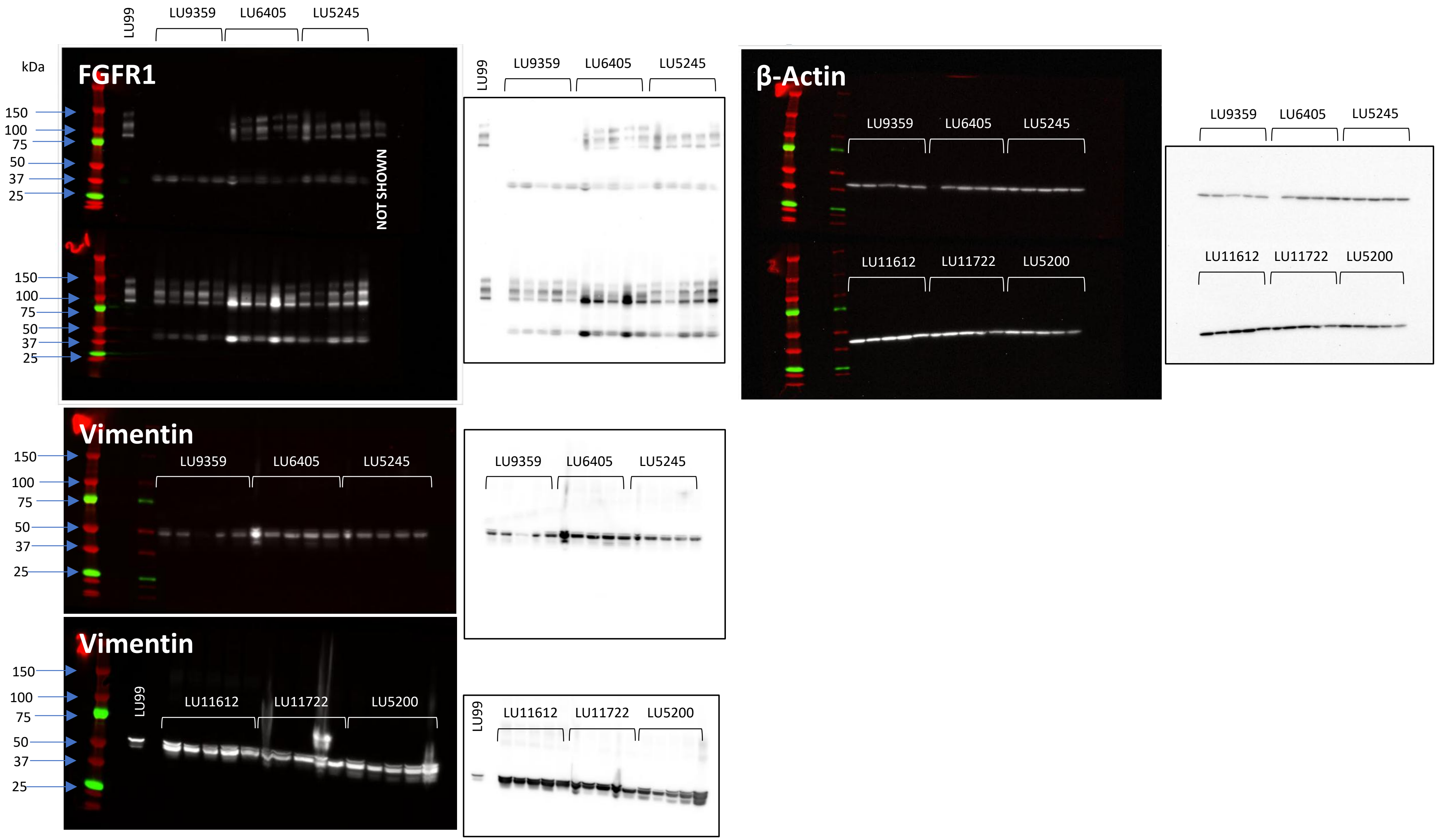

Figure S3: Raw labeled blots for FGFR1, vimentin and β-Actin

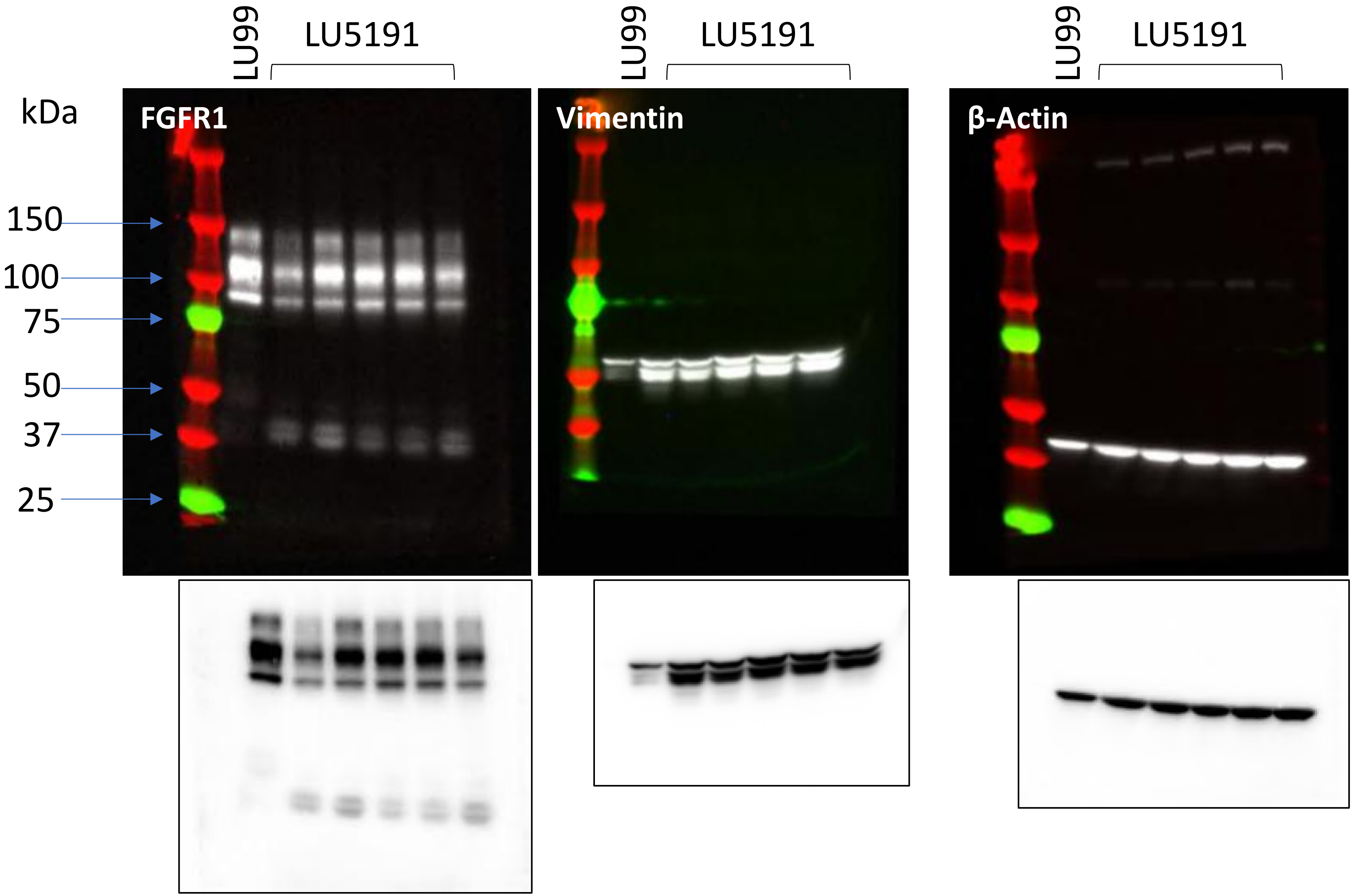

Supplement: S1 Raw Images — (PDF) [file pone.0327588.s005.pdf]
