# Supplementary material for: The FGFR inhibitor pemigatinib overcomes cancer drug resistance to KRAS G12C inhibitors in mesenchymal lung cancer
Source: PLoS One. 2025 Aug 11;20(8):e0327588. doi: 10.1371/journal.pone.0327588 (PMC12338787; doi:10.1371/journal.pone.0327588)
Supplement: S1 Raw Figures — (PPTX) [file pone.0327588.s006.pptx]

## Slide 1
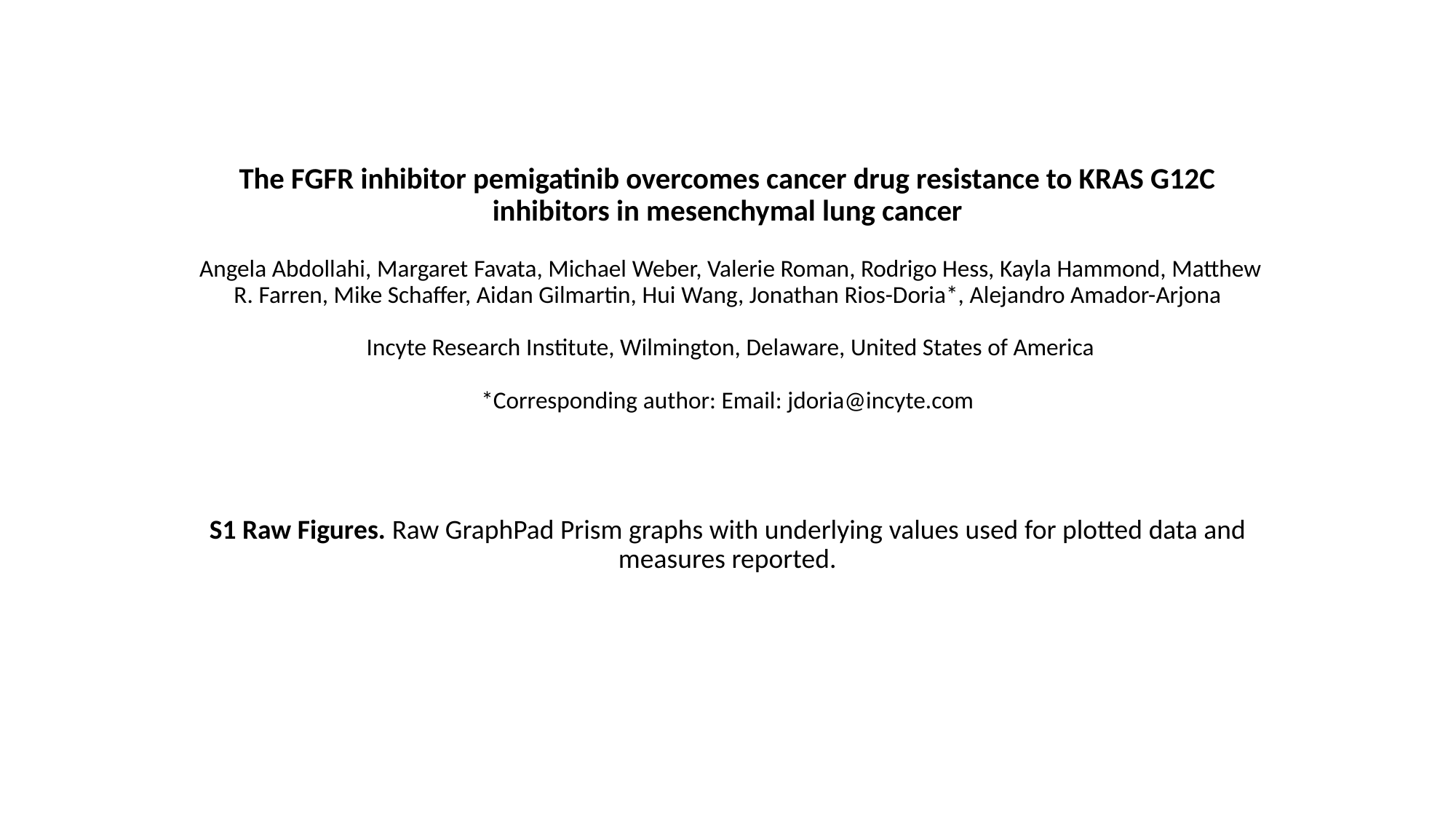

# The FGFR inhibitor pemigatinib overcomes cancer drug resistance to KRAS G12C inhibitors in mesenchymal lung cancer  Angela Abdollahi, Margaret Favata, Michael Weber, Valerie Roman, Rodrigo Hess, Kayla Hammond, Matthew R. Farren, Mike Schaffer, Aidan Gilmartin, Hui Wang, Jonathan Rios-Doria*, Alejandro Amador-Arjona  Incyte Research Institute, Wilmington, Delaware, United States of America   *Corresponding author: Email: jdoria@incyte.com
S1 Raw Figures. Raw GraphPad Prism graphs with underlying values used for plotted data and measures reported.

## Slide 2
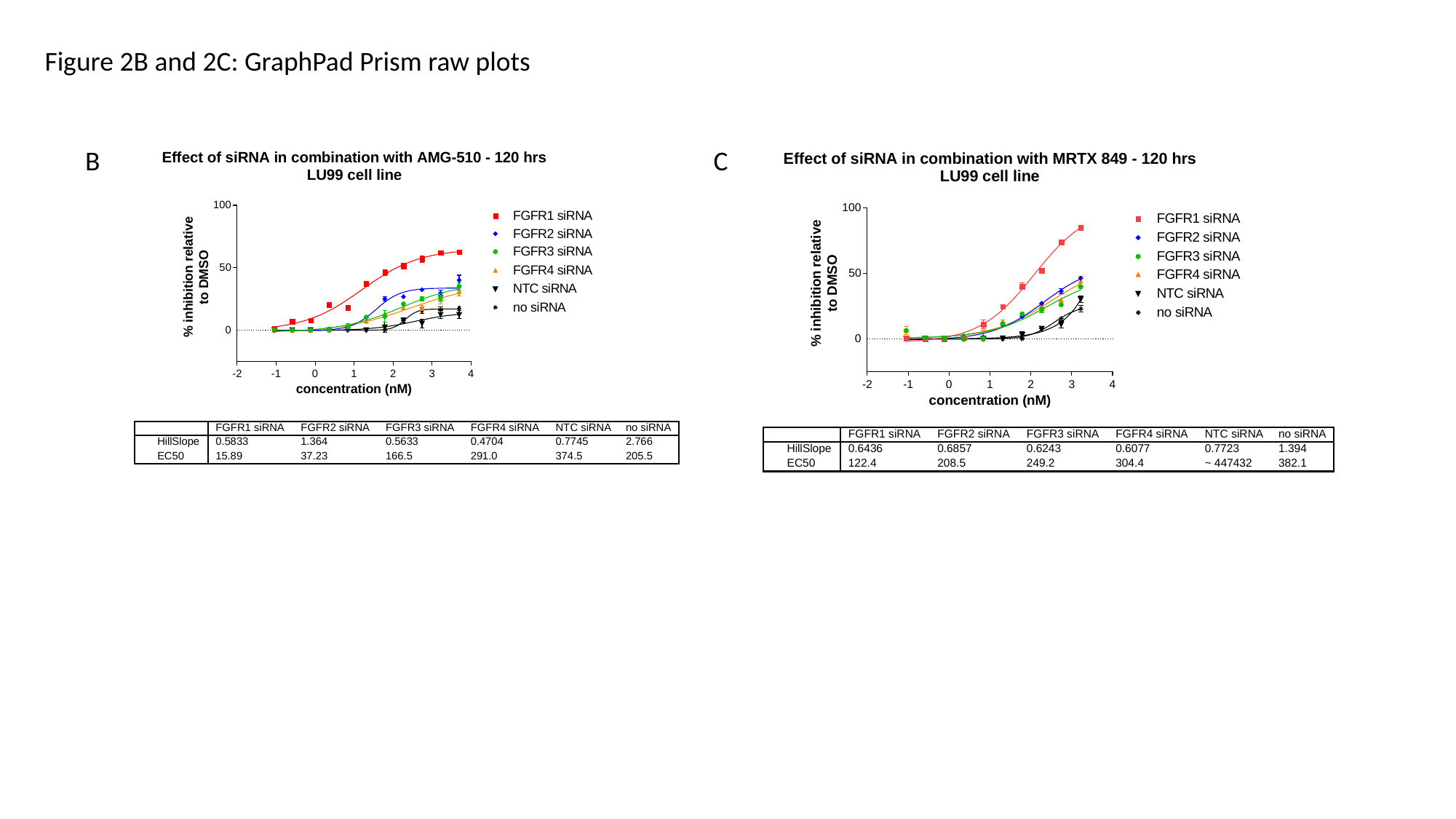

Figure 2B and 2C: GraphPad Prism raw plots
B
C

## Slide 3
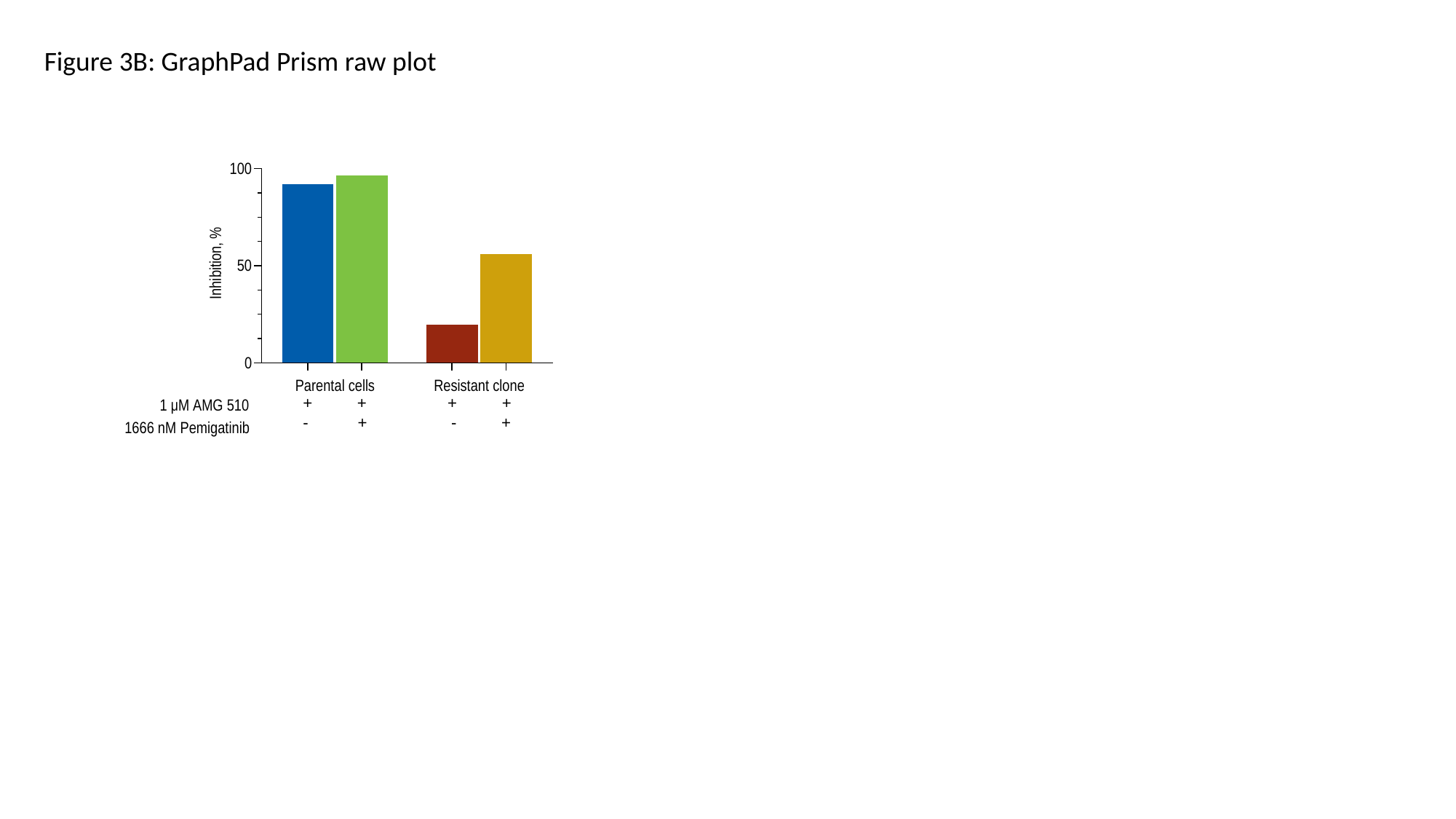

Figure 3B: GraphPad Prism raw plot

## Slide 4
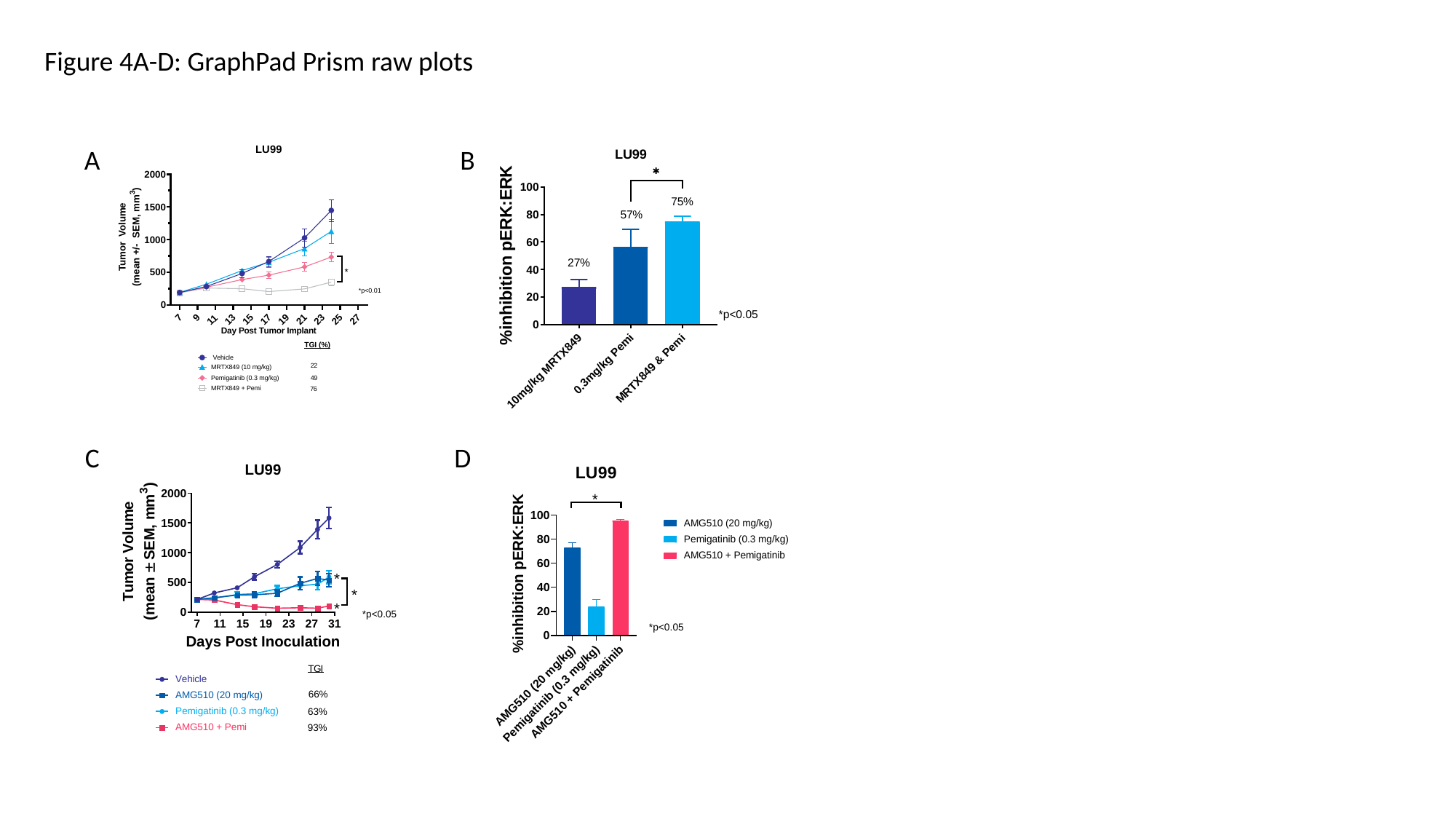

Figure 4A-D: GraphPad Prism raw plots
A
B
C
D

## Slide 5
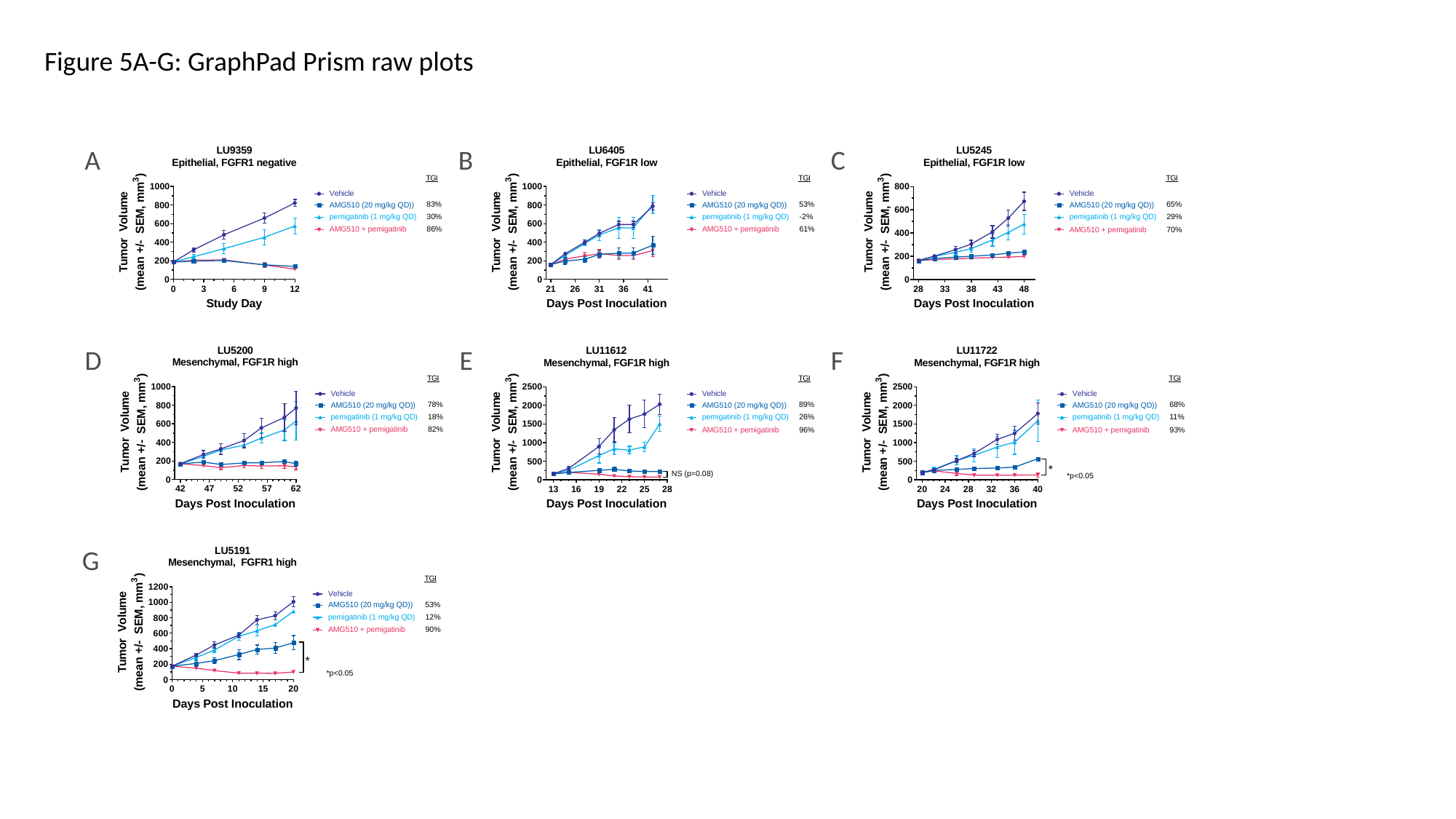

Figure 5A-G: GraphPad Prism raw plots
A
B
C
D
E
F
G
